# Nested TAD hierarchy defines cohesion zones on replicated chromosomes

**DOI:** 10.1038/s44318-026-00902-0

**Published:** 2026-08-17

**Authors:** Zsuzsanna Takacs, Dmitry Mylarshchikov, Sofia Kolesnikova, Christoph C H Langer, Anton Goloborodko, Daniel W Gerlich

**Affiliations:** 1 Institute of Molecular Biotechnology of the Austrian Academy of Sciences (IMBA), Vienna BioCenter (VBC), Dr. Bohr-Gasse 3, 1030 Vienna, Austria; 2 Vienna BioCenter PhD Program, Doctoral School of the University of Vienna and Medical University of Vienna, A-1030 Vienna, Austria

**Keywords:** Cell Cycle, Chromatin, Transcription & Genomics

## Abstract

During each cell cycle, cells must not only duplicate the DNA sequence but also preserve the three-dimensional genome architecture required for gene regulation and DNA repair. After replication, this requires coordination between cohesin-mediated loop extrusion and sister-chromatid cohesion for homology-directed DNA repair. Because extrusion promotes sister resolution whereas cohesion can impede extrusion, how both activities coexist on replicated chromosomes is unclear. Here we show that cohesin function is partitioned within the nested hierarchy of topologically associating domains (TADs): cohesive cohesin accumulates at high-level boundaries to form “cohesion zones,” while loop-extruding cohesin occupies boundaries across all hierarchical levels. Our data support a model in which this segregation emerges from dynamic interplay between cohesin pools and semi-permeable CTCF barriers, which act in cis and in trans to constrain sister–sister misalignment. Thus, a CTCF-defined boundary framework enables sister tethering while preserving dynamic loop folding, allowing replicated genomes to simultaneously support gene regulation and faithful DNA repair.

## Synopsis

How replicated chromosomes coordinate sister chromatid cohesion with dynamic loop folding has been unclear. This study identifies cohesion zones within the nested TAD hierarchy as the organizational principle reconciling these two cohesin functions.

- TAD organization is largely preserved after DNA replication despite establishment of sister chromatid cohesion.
- Cohesive cohesin accumulates selectively at high-level TAD boundaries, forming discrete cohesion zones, whereas loop-forming cohesin occupies boundaries across all hierarchical levels.
- CTCF is required to position cohesion zones and to constrain the genomic range of both cis and trans sister chromatid interactions.
- Loop-extrusion processivity governs how cohesive cohesin is distributed within the TAD hierarchy, tuning the strength and extent of cohesion zones.

## Abstract (web summary)

Opposing cohesin functions are reconciled by cohesive cohesin accumulating at high-level TAD boundaries, while loop-forming cohesin occupies boundaries across all hierarchical levels.

## Graphical abstract

**Figure:**
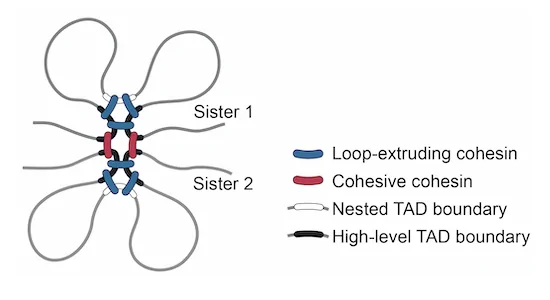

## Introduction

During each cell cycle, proliferating cells duplicate their genomes to pass an identical copy to daughter cells. This requires more than copying the DNA sequence, because replicated sister chromatids must also preserve or re-establish three-dimensional folding and regulatory contact patterns that support genome function. In the G2 phase, sister chromatids remain physically linked for homology-directed DNA repair and faithful chromosome segregation in the subsequent mitosis, while chromatin continues to form dynamic loops that support long-range regulation (Hoencamp and Rowland, 2023; Ochs and Gerlich, 2026). How replicated chromosomes coordinate persistent sister linkage with dynamic 3D folding, however, remains poorly understood.

A central mediator of 3D chromosome organization is cohesin (Michaelis et al, 1997; Guacci et al, 1997), a structural maintenance of chromosomes (SMC) complex with two mechanistically distinct activities. Through ATPase-dependent loop extrusion (Davidson et al, 2019; Kim et al, 2019), cohesin dynamically folds chromatin into loops and topologically associating domains (TADs) (Dixon et al, 2012; Nora et al, 2012; Sexton et al, 2012; Rao et al, 2017; Schwarzer et al, 2017; Wutz et al, 2017; Gassler et al, 2017), thereby regulating long-range enhancer–promoter communication (Robson et al, 2019; Karpinska and Oudelaar, 2023; Buckley et al, 2025). Following DNA replication, a subset of cohesin establishes sister chromatid cohesion by topologically entrapping two DNA molecules (Michaelis et al, 1997; Guacci et al, 1997; Gruber et al, 2003; Haering et al, 2008; Ochs et al, 2024). Cohesion maintains sister loci in proximity to facilitate homology-directed repair of DNA double-strand breaks (Sjögren and Nasmyth, 2001; Sonoda et al, 2001; Ström et al, 2004; Teloni et al, 2025; Phipps and Dubrana, 2022) and provides mechanical stability at centromeres to ensure faithful chromosome segregation in mitosis (Michaelis et al, 1997; Guacci et al, 1997; Tanaka et al, 2000; Oliveira et al, 2010; Nasmyth and Haering, 2009; Yatskevich et al, 2019; Hoencamp and Rowland, 2023; Ochs and Gerlich, 2026). Both functions rely on the same cohesin core complex, which associates with distinct regulatory cofactors to promote either loop extrusion or cohesion. While cohesin’s role in shaping elaborate loop architectures is well established (reviewed in (Yatskevich et al, 2019; Davidson and Peters, 2021; Corsi et al, 2023; Hoencamp and Rowland, 2023; Dekker and Mirny, 2024)), it remains unclear how cohesion is positioned relative to loops and boundary organization on replicated chromosomes.

Because loop extrusion and cohesion act on the same replicated chromosomal template, they might influence each other’s positioning. Loop extrusion folds each sister chromatid, but it also promotes sister chromatid resolution and separation (Goloborodko et al, 2016; Mitter et al, 2020; Batty et al, 2023). Since cohesion requires stable topological entrapment of sister DNAs, extrusion-driven resolution might redistribute cohesive linkages. Conversely, cohesin-mediated linkages can act as barriers to loop extrusion (Chatzidaki et al, 2021; Bastié et al, 2024; Zhao et al, 2025), potentially constraining extrusion-driven resolution. Recent studies in mitotic chromosomes indeed suggest functional coupling between loop extrusion and cohesin engaged in cohesion (Zhao et al, 2025; Samejima et al, 2025). However, mitosis lacks interphase TAD organization and loops are largely formed by condensin-driven extrusion (Naumova et al, 2013; Gibcus et al, 2018), raising the question of how chromosomal context in interphase determines where cohesion can be retained without compromising ongoing loop extrusion.

Here, we combine sister-chromatid–sensitive chromosome conformation mapping with quantitative profiling of cohesin localization, together with acute perturbations of extrusion and cohesion regulators. This integrated strategy allowed us to define the spatial principles that coordinate cohesin’s dual activities across replicated chromosomes, enabling persistent sister linkage while preserving dynamic loop-based folding.

## Results

### Cohesion integrates into a pre-existing 3D genome architecture

**Figure EV1:**
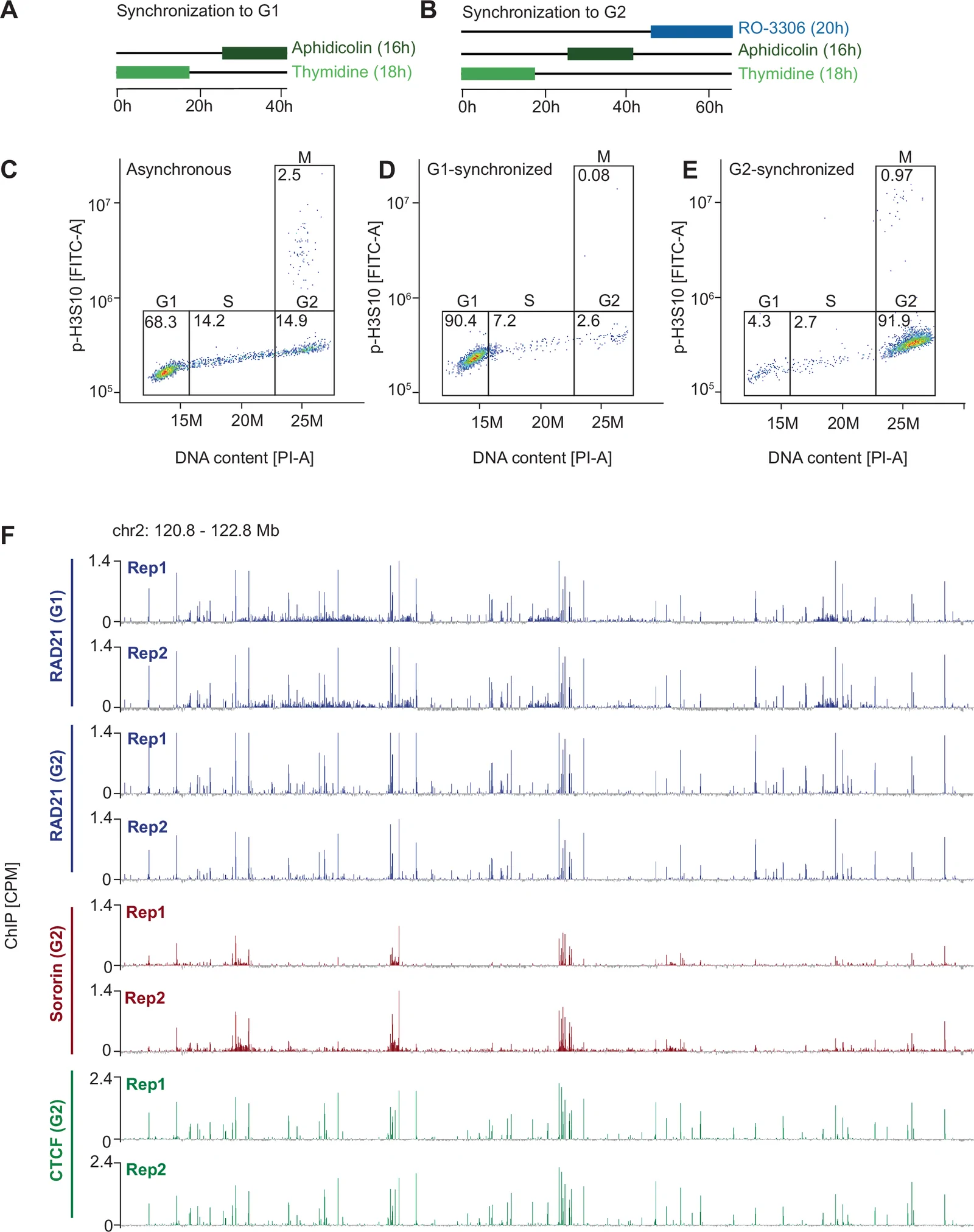
Characterization of cohesin distribution in G1 and G2 cell cycle phases. (**A**,** B**) Schematic of cell synchronization to G1 (**A**) and G2 (**B**) cell cycle phases. (**C**–**E**) Representative cell cycle profiles (phospho-H3S10 vs. DNA content) of asynchronous (**C**), G1-synchronized (**D**), and G2-synchronized (**E**) wild-type HeLa cells. The fraction of cells in the different cell cycle stages is shown. (**F**) RAD21 ChIP-Seq signal tracks (1 kb resolution) from G1-synchronized wild-type HeLa cells and RAD21, Sororin and CTCF ChIP-Seq tracks from G2-synchronized wild-type HeLa cells at the locus shown in Fig. 1A. Individual biological replicates are shown. RAD21 G1- and G2-synchronized replicates were downsampled to a matching sequencing depth (60 mln). ChIP-Seq data are CPM-normalized and centered by subtracting the genome-wide mean.

**Figure 1:**
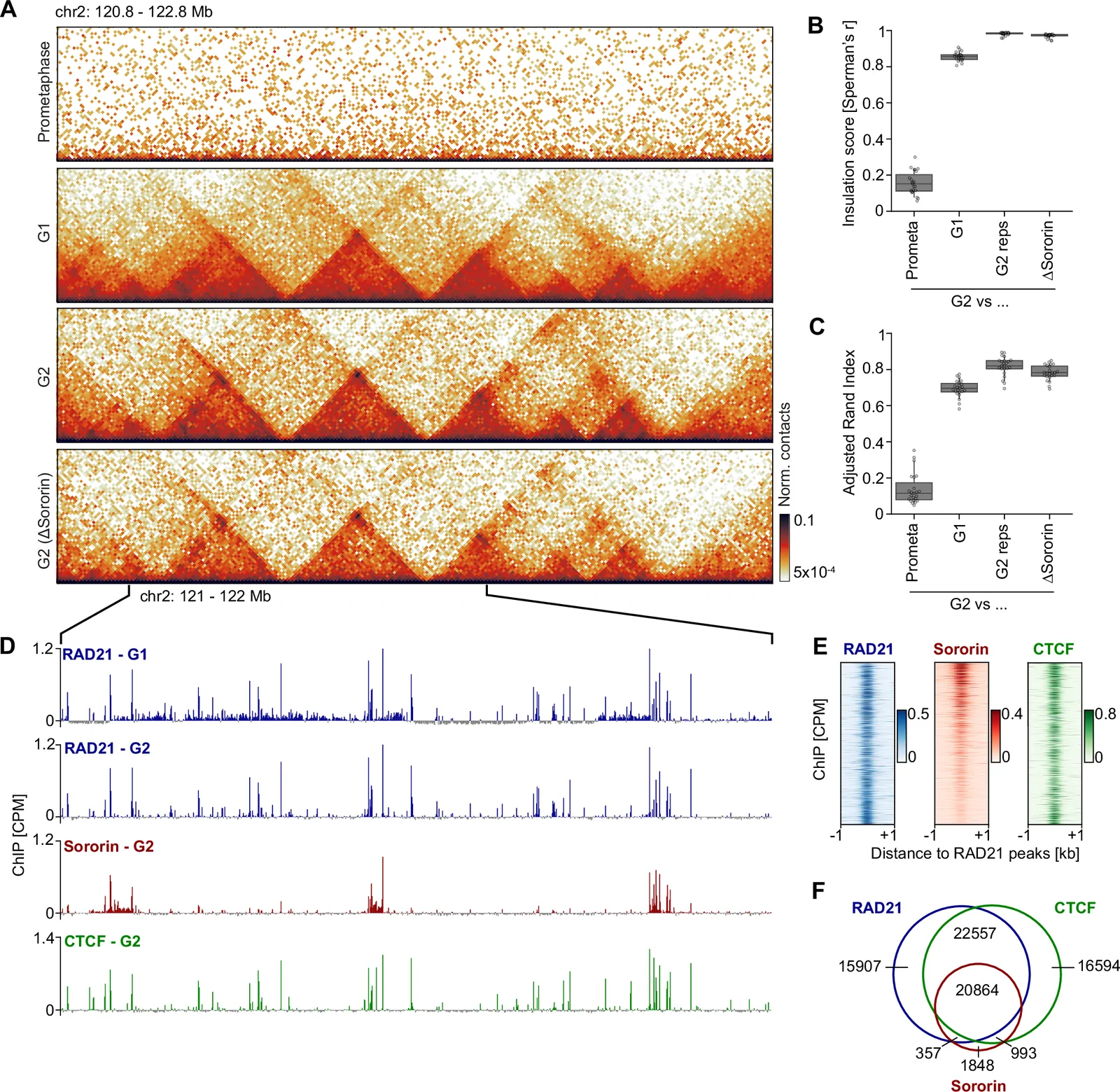
Cohesion integrates into a pre-existing 3D genome landscape. (**A**) Hi-C interaction maps (10 kb resolution, 750 kb separation) from wild-type HeLa cells synchronized to prometaphase, G1, or G2 phase, and from G2-synchronized Sororin-depleted (ΔSororin) cells. G1 and G2 maps were downsampled (499 million contacts) to match the sequencing depth of the ΔSororin map; the prometaphase map contains 100 million contacts. G1 Hi-C data are merged from *n* = 2 independent experiments. (**B**) Spearman correlation of insulation scores from conditions in (**A**). Scores were calculated from Hi-C contact maps (20 kb resolution) using a ±500 kb window per chromosome and compared to G2-synchronized wild-type samples. Biological replicates of wild-type G2 (G2 reps) samples served as positive controls: two replicates were merged (333 million contacts), and the third biological replicate was downsampled to match sequencing depth. Box plots show the median (center line), the 25th and 75th percentiles (box), and the 5th and 95th percentiles (whiskers). (**C**) Adjusted Rand Index of TAD annotations (20 kb resolution, 2 Mb maximum separation) calculated per chromosome. Samples were compared as in (**B**) Box plots show the median (center line), the 25th and 75th percentiles (box), the 5th and 95th percentiles (whiskers). (**D**) ChIP-Seq signal tracks (1 kb resolution) of RAD21 from G1-synchronized HeLa cells and RAD21, Sororin and CTCF from G2-synchronized HeLa cells. A zoomed region corresponding to the area in (**A**) is shown. RAD21 G1 and G2 tracks were downsampled to matching sequencing depths (160 million). (**E**) Stacked line profiles of RAD21, Sororin and CTCF ChIP-Seq signal (50 bp resolution) at ±1 kb regions centered on RAD21 peaks identified in G2-synchronized HeLa cells. Peaks are sorted by the Sororin/RAD21 CPM ratio. (**F**) Venn diagram showing peak overlaps between RAD21, CTCF and Sororin in G2-synchronized wild-type HeLa cells. Reproducible peaks were identified from independent experiments. ChIP-Seq data are CPM-normalized, centered by subtracting the genome-wide mean and represent merged data from *n* = 3 (CTCF) or *n* = 2 (Sororin, RAD21) independent experiments. Wild-type G2-synchronized, prometaphase and G2-synchronized ΔSororin scsHi-C data were reanalyzed from (Mitter et al, 2020; Data ref: Mitter et al, 2020a, 2020b, 2020c).

To determine how cohesion affects higher-order chromosome structure, we compared genome-wide chromatin contact distributions before and after replication in human cells. We selected HeLa cells because multiple validated degron-tagged cell lines are available for the acute depletion of cohesin regulators, together with extensive reference datasets on cohesin distribution and chromosome conformation (Batty et al, 2023; Wutz et al, 2017, 2025; Mitter et al, 2020). Hi-C analysis of G1-synchronized cells (Fig. EV1A,C,D) revealed a TAD organization that was very similar to published Hi-C data from G2-synchronized cells (Mitter et al, 2020; Data ref: Mitter et al, 2020a) (Fig. 1A), consistent with prior work on cell-cycle-phased single-cell Hi-C data (Nagano et al, 2017). In contrast, published reference maps from mitotic cells (Mitter *et al*, 2020; Data ref: Mitter et al, 2020b) showed a marked loss of TAD organization (Fig. 1A). Genome-wide quantitative analyses of TAD architectures using insulation score (Dixon et al, 2012) and Adjusted Rand Index (ARI) (Hubert and Arabie, 1985; An et al, 2019) metrics demonstrated a strong correlation between G1 and G2 but not between G2 and mitosis (Fig. 1B,C). These data indicate that chromatin folding in G1 cells is largely preserved after replication, suggesting that cohesive links form without globally disrupting pre-existing 3D genome organization.

If TAD structures are preserved after replication, then cohesive cohesin must integrate into an existing chromatin architecture without reshaping it. To test this directly, we analyzed published Hi-C data from G2 cells in which the cohesion factor Sororin (Rankin et al, 2005; Schmitz et al, 2007; Ladurner et al, 2016; Ochs et al, 2024) was depleted using an auxin-inducible degron system (Mitter et al, 2020; Data ref: Mitter et al, 2020c). Selective loss of Sororin, and thereby of the cohesive cohesin pool, caused no detectable change in TAD organization relative to wild-type cells (Fig. 1A–C). These findings indicate that the establishment and maintenance of cohesion have minimal structural impact on higher-order chromatin folding.

Since chromatin architecture remains largely unchanged after replication, we next examined how cohesin is positioned along the genome after the establishment of cohesion. We performed ChIP-seq for RAD21, a core subunit shared by both loop-extruding and cohesive cohesin complexes (Yatskevich et al, 2019; Hoencamp and Rowland, 2023; Ochs and Gerlich, 2026) and compared binding profiles between cells synchronized to G1 or G2 (Fig. EV1A–E). RAD21 peaks were highly similar between the two cell cycle phases (Figs. 1D and EV1F), indicating that the establishment of cohesion during S phase does not cause major redistribution of cohesin and instead integrates into a preestablished positional framework.

Following replication, about one-third of cohesin complexes mediate cohesion (Gerlich et al, 2006b; Holzmann et al, 2019; Ochs et al, 2024). To examine how this cohesive pool is distributed along chromosomes, we profiled the distribution of Sororin, a subunit contained in cohesive but not loop-extruding cohesin (Peters and Nishiyama, 2012; Ochs et al, 2024; Schmitz et al, 2007; Rankin et al, 2005; Zhao et al, 2025; Ladurner et al, 2016), using ChIP-seq in G2-synchronized cells (Fig. EV1B,E). Sororin peaks generally co-localized with RAD21 peaks, yet many RAD21 peaks displayed little or no detectable Sororin enrichment, even at loci with high RAD21 occupancy (Figs. 1D,E and EV1F). Overall, only 36% of RAD21 peaks in G2 overlapped with Sororin peaks (Fig. 1F), consistent with previous studies showing that Sororin-bound cohesin localizes to a distinct subset of cohesin sites (Ladurner et al, 2016; Ochs et al, 2024). These data demonstrate that cohesive cohesin is primarily recruited to existing loop-forming sites rather than to new genomic positions, and that its abundance relative to loop-forming cohesin varies substantially along the genome.

To understand what determines which cohesin sites acquire a cohesive function, we investigated the spatial relationship of RAD21 and Sororin with the major cohesin-targeting protein CCCTC-binding factor (CTCF) (Wendt et al, 2008; Parelho et al, 2008; Busslinger et al, 2017; Nora et al, 2017; Wutz et al, 2017; Davidson and Peters, 2021; Hoencamp and Rowland, 2023; Yatskevich et al, 2019; Corsi *e*t al, 2023; Dekker and Mirny, 2024). ChIP-seq of G2-synchronized cells (Fig. EV1B,E) showed that although 91% of Sororin peaks colocalize with CTCF, 37% of CTCF peaks contain RAD21 but lack detectable Sororin (Figs. 1D–F and EV1F). These findings suggest that CTCF is necessary but not sufficient for the selective targeting of cohesive cohesin.

Together, these results show that cohesion integrates into the pre-existing cohesin landscape without substantially reshaping overall TAD organization. Notably, cohesive cohesin accumulates at only a subset of loop-forming cohesin sites, revealing a divergent genomic distribution of the two cohesin pools. Because CTCF occupancy alone does not account for this selectivity, these findings raise questions about what other organizational principles might determine where cohesive cohesin is positioned on replicated chromosomes.

### Nested TAD hierarchy defines split and linked loops

**Figure 2:**
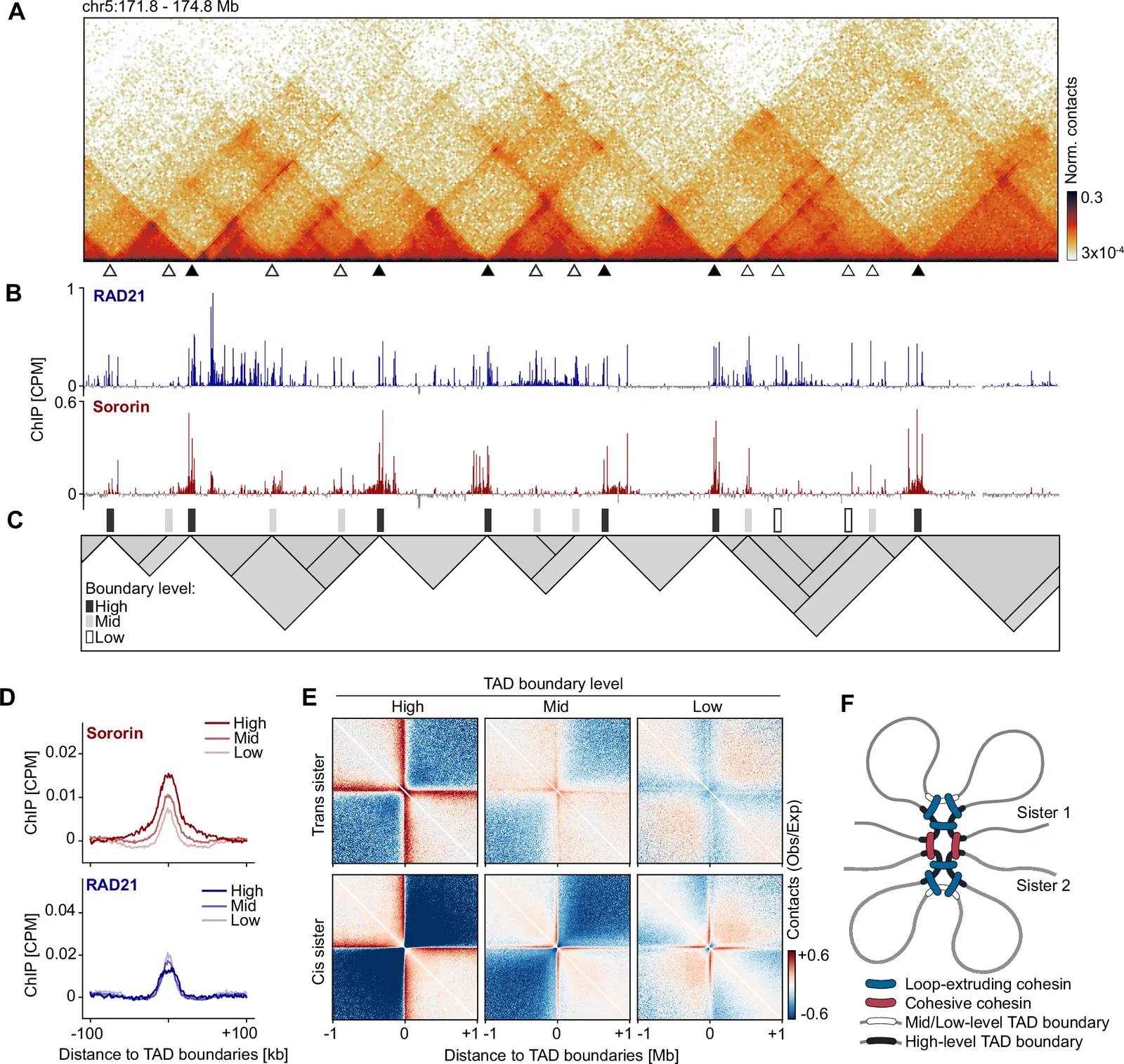
Nested TAD hierarchy defines split and linked loops. (**A**) Representative Hi-C contact map (10 kb resolution, 1.5 Mb separation) from wild-type G2-synchronized HeLa cells. Open arrowheads: TAD boundaries with low/moderate Sororin enrichment; filled arrowheads: TAD boundaries with high Sororin enrichment. (**B**) ChIP-Seq signal tracks (2 kb resolution) of RAD21 (blue) and Sororin (red) from wild-type G2-synchronized HeLa cells at the region shown in (**A**). (**C**) TAD hierarchy annotation generated by OnTAD. Boundaries are classified into high-, mid-, and low-level categories. TAD boundaries were defined at 10 kb resolution and expanded to 20 kb for visualization. (**D**) Average profiles of Sororin (upper panel) and RAD21 (lower panel) ChIP-Seq signal (1 kb resolution) from wild-type G2-synchronized HeLa cells at ±100 kb regions centered at high-, mid- and low-level boundaries. (**E**) Average maps of trans sister (upper row) and cis sister (lower row) scsHi-C contacts (10 kb resolution) from wild-type G2-synchronized HeLa cells at ±1 Mb regions centered at high-, mid-, and low-level boundaries, normalized to genome-wide expected contact frequencies. (**F**) Model depicting sister chromatid organization as defined by TAD hierarchy. ChIP-Seq data are CPM-normalized, centered by subtracting the genome-wide mean and represent merged data from *n* = 2 independent experiments. Wild-type G2-synchronized scsHi-C data were reanalyzed from (Mitter et al, 2020; Data ref: Mitter et al, 2020a).

To investigate how features of higher-order chromosome folding contribute to the selective enrichment of cohesive cohesin along replicated chromosomes, we examined the relationship between TAD architecture and the distribution of cohesin complexes across larger genomic regions in G2-synchronized wild-type cells. Comparisons of chromatin contact maps (Mitter et al, 2020; Data ref: Mitter et al, 2020a) with RAD21 and Sororin ChIP-seq profiles showed that Sororin was primarily enriched at TAD boundaries, whereas many RAD21 peaks inside TAD bodies showed little or no Sororin signal (Fig. 2A,B). Notably, Sororin enrichment was not uniform across boundaries: while RAD21 broadly marked all boundaries (Fig. 2A,B, arrowheads), Sororin concentrated much more prominently at boundaries separating large, high-level domains (Fig. 2A,B, filled arrowheads). In contrast, boundaries delimiting less prominent, nested TADs showed little or no Sororin enrichment (Fig. 2A,B, open arrowheads). These observations suggest that the hierarchy of a boundary within the nested TAD architecture may influence its ability to recruit cohesive cohesin.

**Figure EV2:**
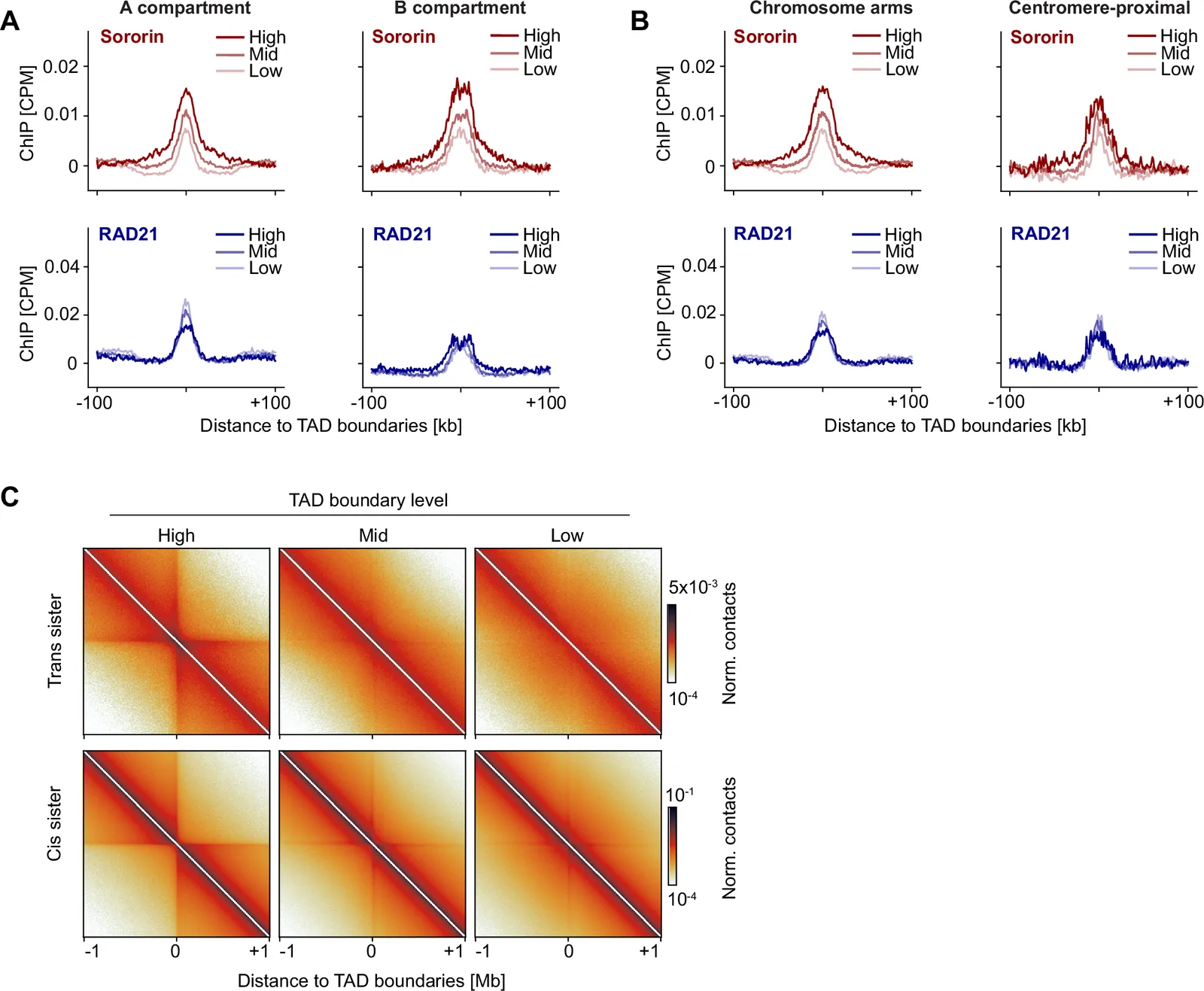
Trans sister contacts accumulate at high-level boundaries. (**A**) Average profiles of Sororin (upper row) and RAD21 (lower row) ChIP-Seq signal (1 kb resolution) at ±100 kb regions centered at high-, mid- and low-level boundaries within A (left column) and B compartments (right column). (**B**) Average profiles of Sororin (upper row) and RAD21 (lower row) ChIP-Seq signal (1 kb resolution) at ±100 kb regions centered at high-, mid- and low-level boundaries on chromosome arms (left column) and near centromeres (right column). (**C**) Average maps of trans sister (upper row) and cis sister (lower row) scsHi-C contacts (10 kb resolution) at ±1 Mb regions centered at high-, mid-, and low-level boundaries. Wild-type G2-synchronized HeLa scsHi-C data were reanalyzed from (Mitter et al, 2020; Data ref: Mitter et al, 2020a).

To systematically evaluate this relationship, we assigned hierarchy levels to TAD boundaries using OnTAD (An et al, 2019), which identifies nested domains and stratifies TADs according to their position within the TAD hierarchy (Fig. 2C). Based on the hierarchy of corresponding TADs, we classified boundaries into high-, mid-, and low-level categories (Fig. 2C) and analyzed ChIP-seq signals across these groups. Aggregate profiles revealed that Sororin was significantly enriched at high-level boundaries compared to mid- and low-level ones (Fig. 2D, upper panel), whereas RAD21 was more evenly distributed, displaying even modest enrichment at lower-level boundaries (Fig. 2D, lower panel). The increased levels of Sororin at high-level boundaries compared to low-level boundaries were observed both in A- and B-type chromatin compartments (Fig. EV2A), as well as centromere-proximal chromosome regions (Fig. EV2B). These data demonstrate that while loop-forming cohesin broadly associates with boundaries, cohesive cohesin preferentially localizes to the top-level boundaries within the nested TAD hierarchy.

To directly examine how loops and sister-chromatid linkages are organized within the TAD hierarchy, we used sister-chromatid-sensitive Hi-C (scsHi-C). This approach distinguishes contacts within a chromatid (cis sister contacts) from contacts between sister chromatids (trans sister contacts) through 4-thiothymidine labeling of nascent DNA (Mitter et al, 2020, 2022). Although previous work reported that trans sister-chromatid contacts are enriched at TAD boundaries, our cohesin ChIP-seq data suggest a more complex distribution within the nested TAD hierarchy. To investigate this, we reanalyzed published scsHi-C data from G2-synchronized cells (Mitter et al, 2020; Data ref: Mitter et al, 2020a) to generate aggregate maps centered on TAD boundaries stratified by their position within the hierarchy. Trans sister contacts were enriched in stripes emanating from high-level boundaries, but not at mid-level boundaries, and were even depleted at low-level boundaries relative to the genome-wide average (Figs. 2E, upper row and EV2C, upper row). These results indicate that high-level TAD boundaries act as discrete cohesion hubs.

To understand how the variability in trans sister contact density across the boundary hierarchy relates to the loop architectures within sister chromatids, we next analyzed cis sister contact patterns. Average contact maps aligned to TAD boundaries revealed prominent contact enrichment stripes across all hierarchy levels, reflecting local loop formation (Figs. 2E, lower row and EV2C, lower row). While these stripes spanned a greater genomic range at high-level boundaries, they remained prominent at mid- and low-level boundaries as well. These results reveal a marked contrast between inter- and intra-sister organization: whereas trans sister contact enrichment is confined to high-level boundaries, cis sister looping occurs at all hierarchical levels. This difference matches the distribution of RAD21 binding observed by ChIP-seq, where cohesin broadly marks boundaries across the hierarchy while cohesive cohesin is restricted to the strongest ones.

Together, these observations show a functional distinction within the TAD hierarchy: mid- and low-level boundaries primarily form loop structures independently on each sister chromatid, whereas high-level boundaries establish loops and at the same time function as specialized cohesion zones (Fig. 2F). This hierarchical TAD organization folds replicated chromosomes into a nested architecture in which small loops form separately on each sister chromatid, while sister chromatids are linked at the anchors of large loops.

### CTCF restricts the misalignment of sister chromatids

**Figure EV3:**
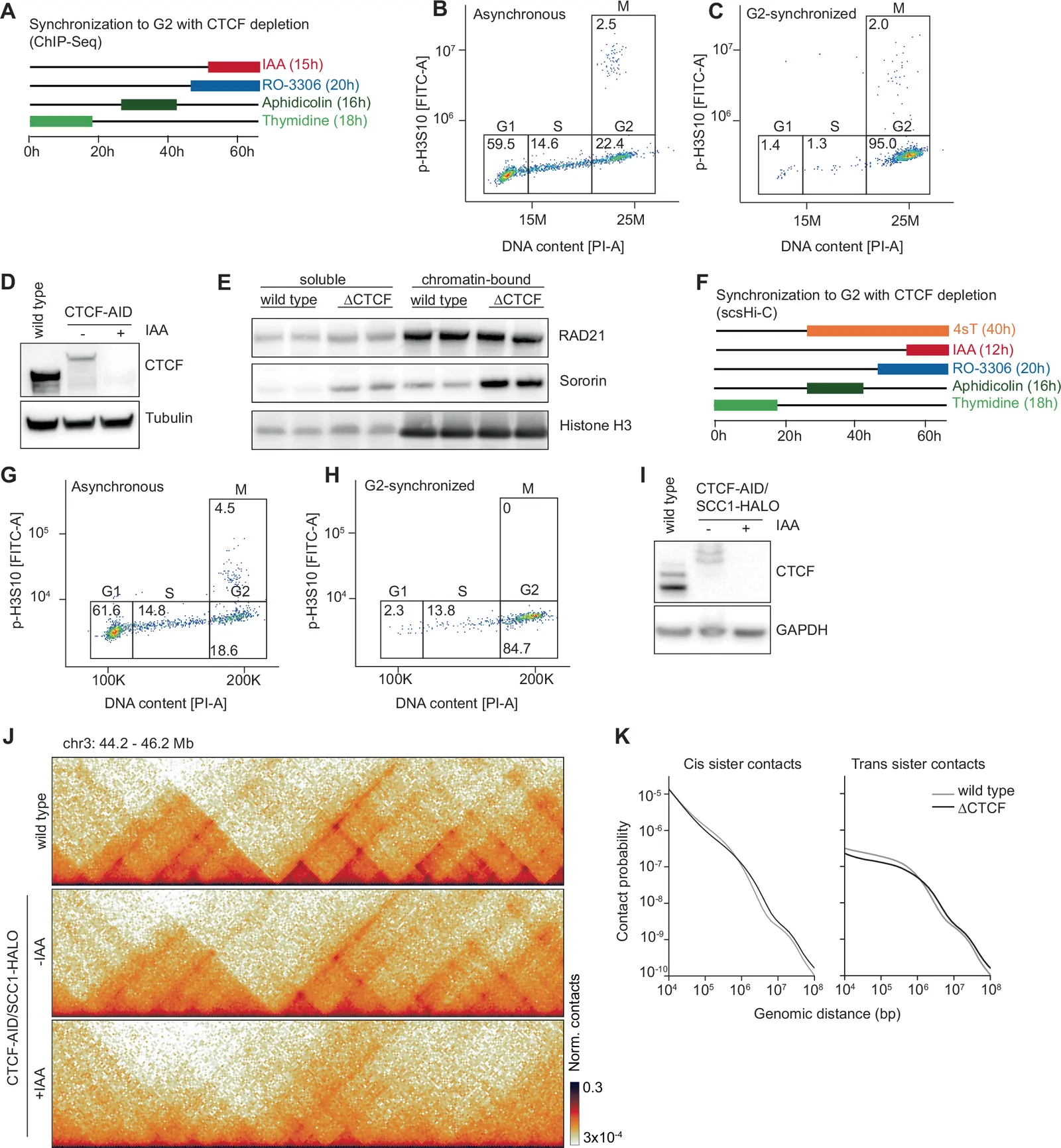
Characterization of CTCF depletion for scsHi-C and ChIP-Seq experiments. (**A**) Schematic of G2 cell synchronization and CTCF depletion for ChIP-Seq. (**B**, **C**) Representative cell cycle profiles (phospho-H3S10 vs. DNA content) of asynchronous (**B**) and G2-synchronized (**C**) HeLa CTCF-AID cells corresponding to (**A**). The fraction of cells in the different cell cycle stages is shown. (**D**) Western blot confirming CTCF depletion upon indole-3-acetic acid (IAA) treatment in G2-synchronized CTCF-AID cells corresponding to (**A**–**C**). Tubulin serves as a loading control. (**E**) Western blot confirming the presence of RAD21 and Sororin on chromatin in G2-synchronized CTCF-depleted (ΔCTCF, CTCF-AID) cells, as shown in (**A**, **C**). Histone H3 serves as a loading control. Data were representative of *n* = 2 independent experiments. (**F**) Schematic of G2 cell synchronization and CTCF depletion for scsHi-C. (**G**,** H**) Representative cell cycle profiles (phospho-H3S10 vs. DNA content) of asynchronous (**G**) and G2-synchronized (**H**) CTCF-AID/SCC1-HALO cells corresponding to (**F**). The fraction of cells in the different cell cycle stages is shown. (**I**) Western blot confirming CTCF depletion upon indole-3-acetic acid (IAA) treatment in G2-synchronized CTCF-AID/SCC1-HALO cells corresponding to (**F**–**H**). GAPDH serves as a loading control. (**J**) Representative Hi-C contact maps (10 kb resolution, 1 Mb separation) from wild-type cells, and CTCF-AID/SCC1-HALO cells in the presence and absence of indole-3-acetic acid (IAA). (**K**) Distance-dependent contact probability (P(s)) curves for cis and trans sister scsHi-C-contacts from G2-synchronized wildtype and CTCF-depleted (ΔCTCF, CTCF-AID/SCC1-HALO) cells. scsHi-C data from G2-synchronized CTCF-AID/SCC1-HALO cells without target depletion (-IAA) and upon CTCF depletion (ΔCTCF, +IAA) are merged from *n* = 3 technical replicates and *n* = 5 independent experiments, respectively. Wild-type G2-synchronized scsHi-C data were reanalyzed from (Mitter et al, 2020; Data ref: Mitter et al, 2020a).

**Figure 3:**
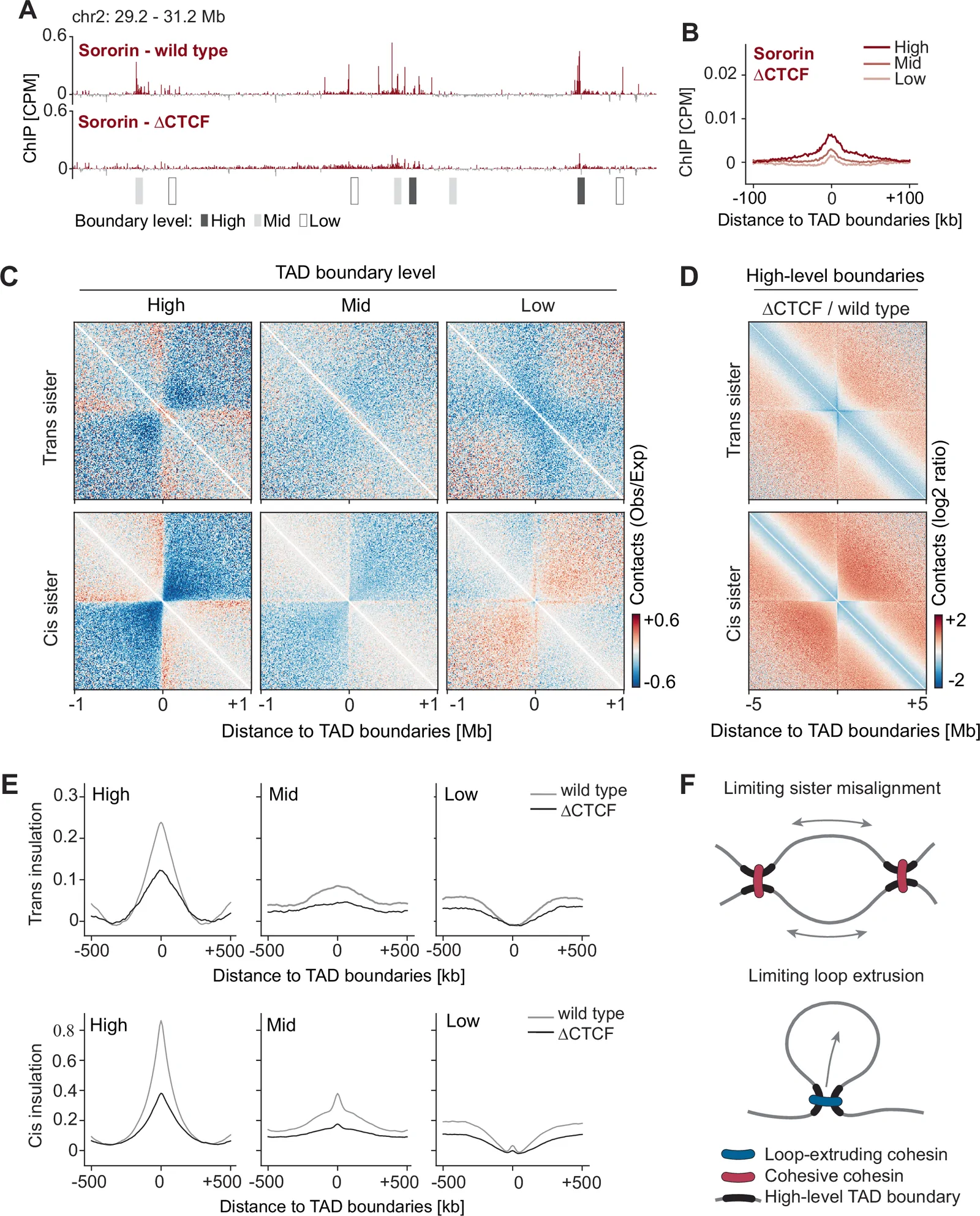
CTCF positions cohesive cohesin and restricts sister chromatid misalignment. (**A**) Representative Sororin ChIP-Seq signal track (2 kb resolution) from G2-synchronized wild-type HeLa and CTCF-depleted (ΔCTCF and CTCF-AID) cells with TAD boundary annotations (defined at 10 kb; expanded to 20 kb for visualization) below. (**B**) Average profiles of Sororin ChIP-Seq signal (1 kb resolution) from G2-synchronized ΔCTCF (CTCF-AID) cells at ±100 kb regions centered at high-, mid- and low-level boundaries. (**C**) Average maps of trans sister (upper row) and cis sister (lower row) scsHi-C contacts (10 kb resolution) at ±1 Mb regions centered at high-, mid-, and low-level boundaries from G2-synchronized ΔCTCF (CTCF-AID/SCC1-HALO) cells normalized to genome-wide expected contact frequencies. (**D**) Ratio pileups (ΔCTCF/wildtype) of trans sister (upper panel) and cis sister (lower panel) scsHi-C contacts (20 kb resolution) at ±5 Mb regions centered at high-level boundaries. Ratios represent contacts from CTCF-depleted conditions (ΔCTCF) normalized over wild-type conditions. (**E**) Average insulation score profiles calculated from G2-synchronized trans sister (upper row) and cis sister (lower row) contacts (10 kb resolution) in wildtype (gray line) and ΔCTCF (CTCF-AID/SCC1-HALO) cells (black line) at ±500 kb regions centered at high-, mid-, and low-level boundaries. (**F**) Model illustrating CTCF’s role in limiting sister chromatid misalignment and loop extrusion in cis. ChIP-Seq data are CPM-normalized, centered by subtracting the genome-wide mean and represent merged data from *n* = 2 independent experiments. scsHi-C data for ΔCTCF are merged from *n* = 5 independent experiments. Wild-type G2-synchronized scsHi-C data were reanalyzed from (Mitter et al, 2020; Data ref: Mitter et al, 2020a).

Given that cohesion is concentrated at high-level TAD boundaries, we asked which molecular factors specify this organization. Because CTCF positions loop-forming cohesin at TAD boundaries (Wendt et al, 2008; Parelho et al, 2008; Rao et al, 2014; Fudenberg et al, 2016; Sanborn et al, 2015; Busslinger et al, 2017; Nora et al, 2017; Wutz et al, 2017), we tested whether CTCF also positions cohesion zones. We therefore depleted CTCF in G2-synchronized cells (CTCF-AID) using an auxin-inducible degron system (AID1) (Fig. EV3A–D) (Nishimura et al, 2009). Because untreated CTCF-AID cells showed modest basal degradation (Fig. EV3D) (Yesbolatova et al, 2020), we compared depletion conditions to wildtype. To determine how CTCF contributes to the positioning of cohesive cohesin, we profiled Sororin binding by ChIP-seq relative to TAD boundary positions defined in wild-type cells. CTCF depletion strongly reduced Sororin enrichment at TAD boundaries, including high-level boundaries (Fig. 3A,B), whereas Western blotting showed that chromatin-bound RAD21 and Sororin levels remained high (Fig. EV3E). Thus, CTCF is dispensable for global chromatin association of cohesive cohesin but required for its focal enrichment at the high-level TAD boundaries that define cohesion zones.

Given that CTCF is required to concentrate Sororin at TAD boundaries, we next asked how this positioning impacts sister-chromatid organization. To address this, we depleted CTCF in G2-synchronized cells (CTCF-AID/SCC1-HALO) (Fig. EV3F–I). Because untreated cells showed modest basal CTCF degradation (Fig. EV3I) accompanied by a modest chromatin-organization phenotype (Fig. EV3J), depletion conditions were compared with wild-type cells. Depleting CTCF strongly reduced TAD structures in conventional Hi-C maps (Fig. EV3J), consistent with CTCF’s established role in boundary positioning of loop-based architectures. We then compared scsHi-C maps from wildtype (Mitter et al, 2020; Data ref: Mitter et al, 2020a) and CTCF-depleted cells to resolve sister-specific interactions. Upon CTCF depletion, boundary-associated contact stripes were nearly abolished in trans sister maps (Fig. 3C, upper row), indicating that boundary-centered sister–sister organization depends on CTCF. Cis sister maps likewise showed a marked loss of boundary-associated stripes, in line with previous reports (Wutz et al, 2017; Nora et al, 2017) (Fig. 3C, lower row). Together, these data show that CTCF not only positions loops within each chromatid but also organizes boundary-associated interactions between sister chromatids.

The displacement of cohesive cohesin away from TAD boundaries in CTCF-depleted cells raised the question of whether this redistribution affects trans sister interactions beyond boundary coordinates. To address this, we calculated ratios of contact densities in CTCF-depleted cells relative to wild-type cells in a larger genomic window. Aggregate maps of trans sister contacts centered on high-level TAD boundaries revealed a pronounced redistribution from short-range to longer-range interactions at the boundary and at adjacent genomic regions (Fig. 3D, upper panel). Cis sister contacts showed a similar shift toward longer genomic distances (Fig. 3D, lower panel). Genome-wide P(s) curves consistently shifted from short- to long-range contacts in both cis and trans (Fig. EV3K). Together, these analyses indicate that CTCF constrains the genomic range of interactions within and between sister chromatids.

The constraint imposed by CTCF on long-range genomic interactions is consistent with its well-established function as a barrier to loop extrusion (Fudenberg et al, 2016; Nuebler et al, 2018; Wutz et al, 2017; Nora et al, 2017; Davidson et al, 2023; Chang et al, 2023; Zhang et al, 2023). In conventional Hi-C experiments, such constraints are detected using insulation metrics that quantify contact depletion across domain boundaries. To examine how insulation is organized in replicated chromosomes, we quantified insulation strength across TAD boundaries in cis and trans sister contact maps using the insulation score (Wutz et al, 2017). In wild-type cells, insulation strength scaled with boundary hierarchy: high-level boundaries strongly insulated both cis sister and trans sister contacts, whereas mid- and low-level boundaries showed progressively weaker insulation, with little or no insulation in trans (Fig. 3E, gray lines). CTCF depletion strongly reduced insulation in both cis and trans (Fig. 3E, black lines). Thus, CTCF establishes topological barriers not only for loop interactions within chromatids but also for interactions across sister chromatids, effectively controlling the positioning and the spatial extent of trans sister interactions.

Together, these results establish CTCF as a central regulator of replicated chromosome architecture: by positioning loop-forming cohesin at TAD boundaries and concentrating cohesive cohesin at high-level boundaries CTCF imposes a topological barrier that restricts the genomic range of cis and trans sister interactions (Fig. 3F). By limiting the genomic span of trans sister interactions, CTCF constrains large-scale sister chromatid misalignment while preserving dynamic loop architectures within intervening domains.

### Loop extrusion positions cohesive cohesin within the TAD hierarchy

While our results show that CTCF positions both loop-forming and cohesive cohesin at TAD boundaries, it remains unclear how the two cohesin pools become differentially enriched across the nested TAD hierarchy. One possibility is that this hierarchy-dependent distribution reflects dynamic interactions between the two pools. Loop-forming cohesin is positioned at boundaries through its intrinsic ATP-dependent motor activity (Davidson et al, 2019; Kim et al, 2019), enabling translocation along chromatin until extrusion is stalled at CTCF boundary elements (Fudenberg et al, 2016; Sanborn et al, 2015; Wutz et al, 2017; Nora et al, 2017; Li et al, 2020; Nora et al, 2020; Davidson et al, 2023). In contrast, cohesive cohesin is not known to have a motor activity and it does not contain the NIPBL subunit required for ATP hydrolysis and DNA translocation (Panizza et al, 2000; Sumara et al, 2000; Hartman et al, 2000; Kikuchi et al, 2016; Petela et al, 2018; Ciosk et al, 2000; Murayama and Uhlmann, 2014; Davidson et al, 2019; Kim et al, 2019; Wutz et al, 2025; Shah et al, 2026; Srinivasan et al, 2019; Nasmyth et al, 2023). Its genomic distribution may therefore depend on forces generated by loop-extruding cohesin. Indeed, mitotic studies suggest that loop-extruding cohesin can push cohesive cohesin along DNA toward boundary elements (Zhao et al, 2025). However, how such extrusion-driven pushing would generate the pronounced TAD hierarchy-dependent enrichment of cohesive cohesin observed in interphase remains unclear.

**Figure EV4:**
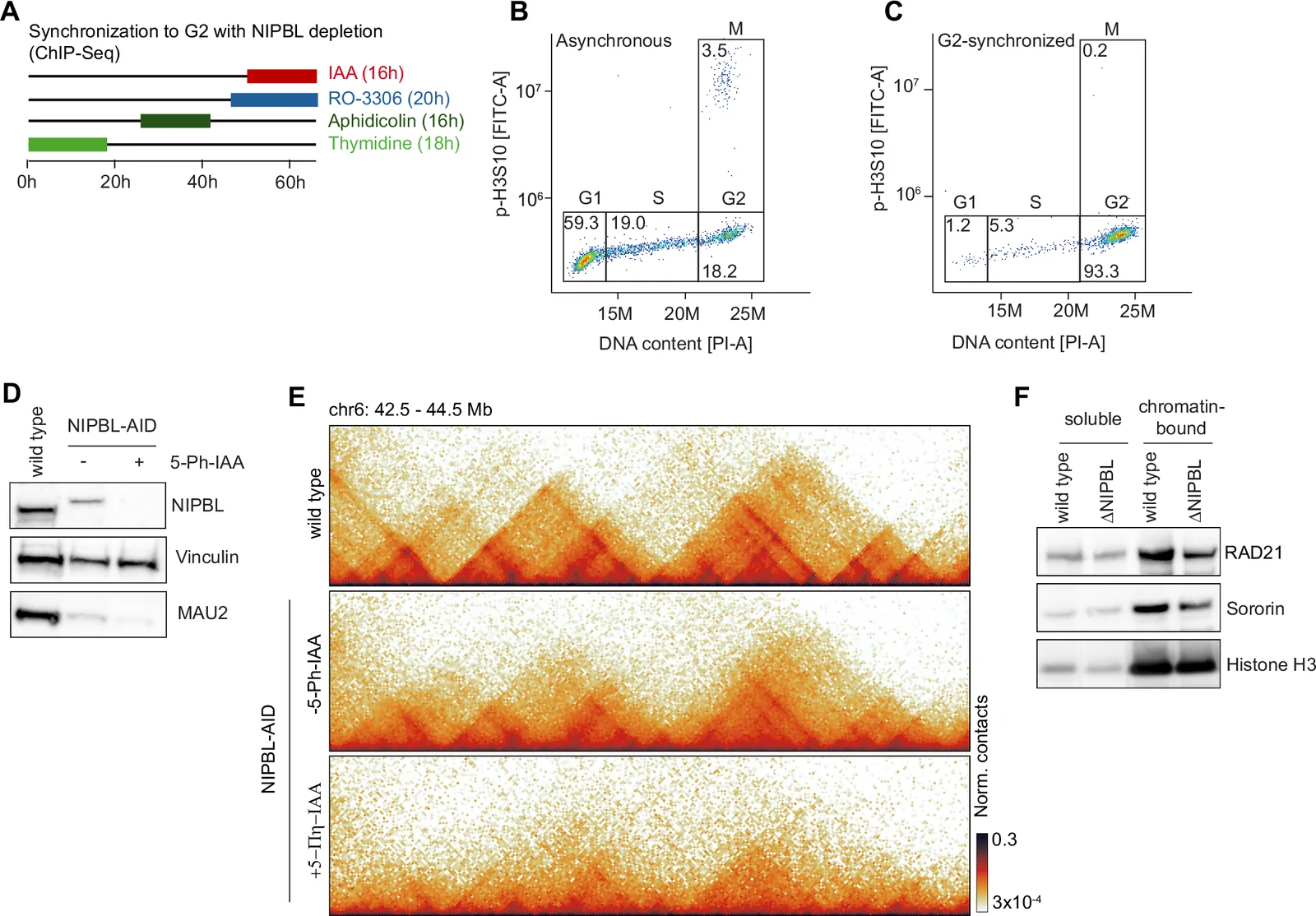
Characterization of NIPBL depletion for ChIP-Seq experiments. (**A**) Schematic of G2 synchronization and NIPBL depletion for ChIP-Seq. (**B**,** C**) Representative cell cycle profiles (phospho-H3S10 vs. DNA content) of asynchronous (**B**) and G2-synchronized (**C**) HeLa NIPBL-AID cells corresponding to (A). The fraction of cells in the different cell cycle stages is shown. (**D**) Western blot confirming NIPBL depletion upon 5-phenyl-indole-3-acetic acid (5-Ph-IAA) treatment in G2-synchronized NIPBL-AID cells corresponding to (**A**–**C**). NIPBL depletion was additionally confirmed by immunoblotting for its binding partner MAU2 (Watrin and Peters, 2006; Banigan et al, 2023). Vinculin serves as a loading control. (**E**) Representative Hi-C contact maps (10 kb resolution, 1 Mb separation) from G2-synchronized wild-type cells, and NIPBL-AID cells in the presence and absence of 5-phenyl-indole-3-acetic acid (5-Ph-IAA). (**F**) Western blot confirming the presence of RAD21 and Sororin on chromatin in G2-synchronized NIPBL-depleted (ΔNIPBL, NIPB-AID) cells, as shown in (**A**, **C**). Histone H3 serves as a loading control. Data are representative of *n* = 2 independent experiments. scsHi-C data from G2-synchronized NIPBL-AID cells without target depletion (-5-Ph-IAA) are merged from *n* = 3 technical replicates. Wild-type G2-synchronized scsHi-C data were reanalyzed from (Mitter et al, 2020; Data ref: Mitter et al, 2020a). ΔNIPBL G2-synchronized scsHi-C data were reanalyzed from (Batty et al, 2023; Data ref: Batty et al, 2023).

**Figure 4:**
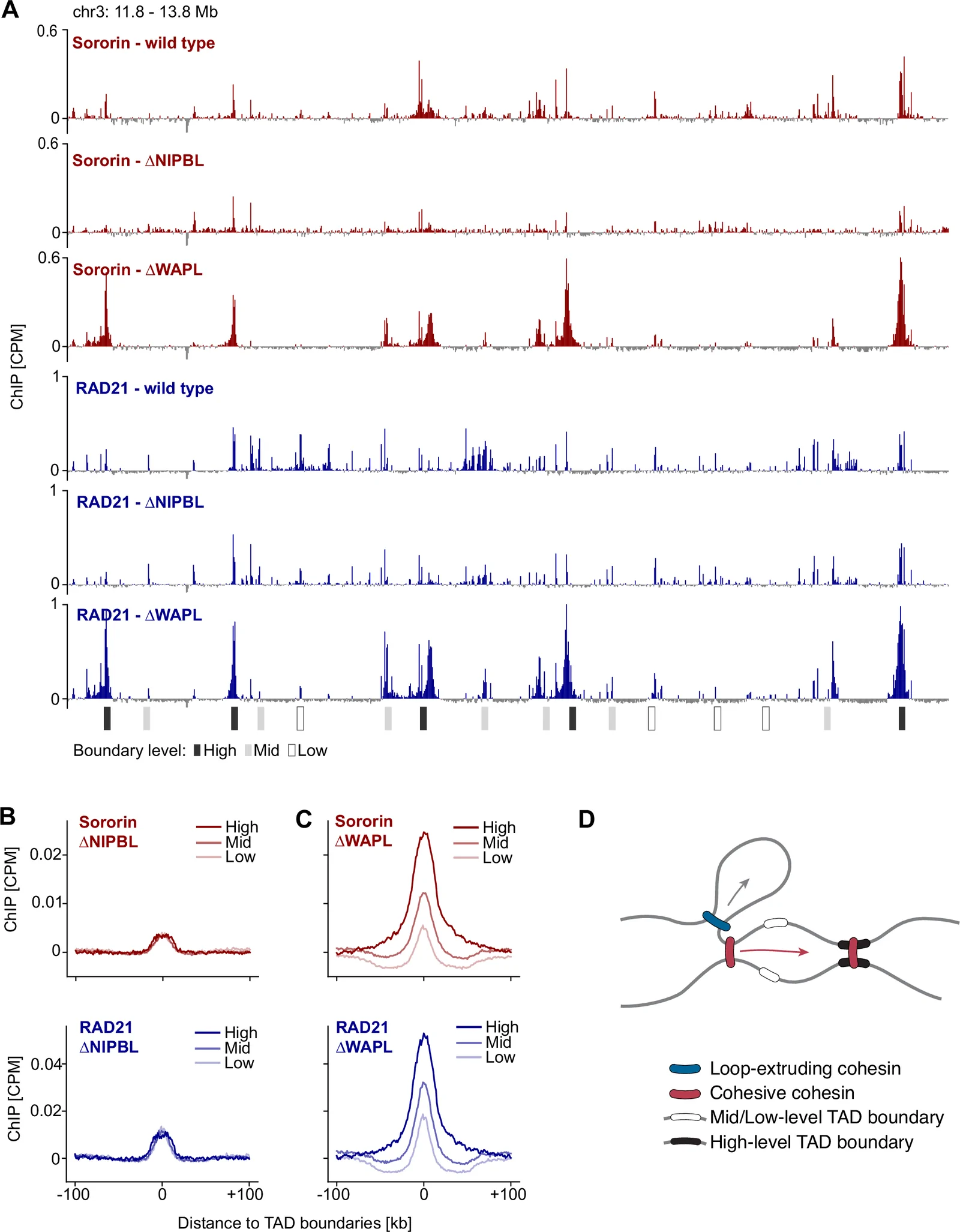
Loop extrusion concentrates cohesive cohesin at high-level TAD boundaries. (**A**) Representative ChIP-Seq tracks (2 kb resolution) of Sororin (red) and RAD21 (blue) from G2-synchronized wildtype, NIPBL-depleted (ΔNIPBL, NIPBL-AID) and WAPL-depleted (ΔWAPL, WAPL-DTAG) cells, with TAD boundary annotations (defined at 10 kb; expanded to 20 kb for visualization) below. (**B**) Average profiles of Sororin (upper panel, red) and RAD21 (lower panel, blue) ChIP-Seq signal (1 kb resolution) from G2-synchronized ΔNIPBL cells at ±100 kb regions centered at high-, mid-, and low-level boundaries. (**C**) Average profiles of Sororin (upper panel, red) and RAD21 (lower panel, blue) ChIP-Seq signal (1 kb resolution) from G2-synchronized ΔWAPL cells at ±100 kb regions centered at high-, mid- and low-level boundaries. (**D**) Model depicting loop extrusion-mediated positioning of cohesive cohesin at strong TAD boundaries. ChIP-Seq data are CPM-normalized, centered by subtracting the genome-wide mean and represent merged data from *n* = 2 independent experiments.

To test whether loop extrusion establishes the differential enrichment of cohesive cohesin across TAD boundaries in interphase, we depleted NIPBL in G2-synchronized cells (NIPBL-AID) using an auxin-inducible degron (AID2) (Fig. EV4A–D) (Yesbolatova et al, 2020). This treatment suppresses loop extrusion while preserving previously established cohesion. Because AID-tagged NIPBL showed modest basal degradation in the absence of auxin (Fig. EV4D), accompanied by a modest effect on chromatin organization (Fig. EV4E), we used wild-type cells as the reference for all comparisons (Mitter et al, 2020; Data ref: Mitter et al, 2020a). Analysis of published Hi-C data upon NIPBL depletion (Batty et al, 2023; Data ref: Batty et al, 2023) showed a marked loss of TAD structures under these conditions (Fig. EV4E). ChIP-seq profiling revealed that the selective enrichment of Sororin at high-level boundaries was lost following NIPBL depletion. Instead, Sororin enrichment was reduced to similarly low levels at high-, mid-, and low-level boundaries, thereby abolishing its hierarchy-dependent distribution (Fig. 4A, red tracks; 4B, upper panel). The reduction in boundary-centered enrichment was accompanied by a slightly elevated Sororin signal between boundaries (Fig. 4A), whereas Western blot analysis showed that chromatin-bound Sororin levels remained high (Fig. EV4F). Together, these data indicate that cohesive cohesin is redistributed from boundary-centered peaks to inter-boundary chromatin rather than dissociating from chromatin. RAD21 similarly showed reduced boundary-centered enrichment and a more uniform genomic distribution (Fig. 4A, blue tracks; B, lower panel). Thus, loop extrusion concentrates cohesive cohesin at TAD boundaries, with the strongest effect at high-level boundaries; in its absence, boundary enrichment is reduced across all hierarchy levels and cohesive cohesin becomes more broadly distributed across inter-boundary chromatin.

**Figure EV5:**
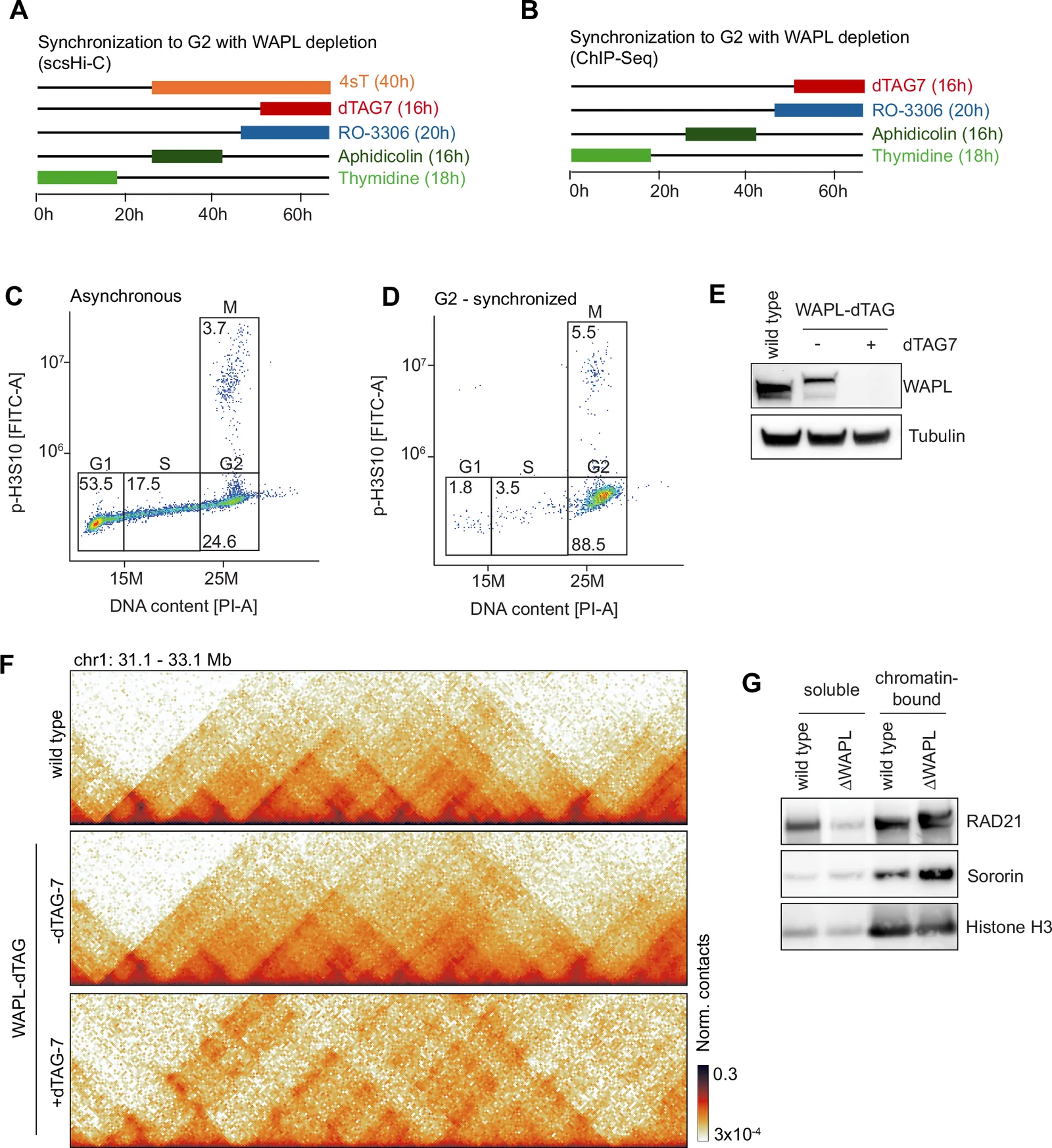
Characterization of WAPL depletion for scsHi-C and ChIP-Seq experiments. (**A**,** B**) Schematic of G2 synchronization and WAPL depletion for scsHi-C (**A**) and ChIP-Seq (**B**). (**C**, **D**) Representative cell cycle profiles (phospho-H3S10 vs. DNA content) of asynchronous (**C**) and G2-synchronized (**D**) HeLa WAPL-dTAG cells corresponding to (**A**, **B**). The fraction of cells in the different cell cycle stages is shown. (**E**) Western blot confirming WAPL depletion upon dTAG-7 treatment in G2-synchronized WAPL-dTAG cells corresponding to (**A**, **B**, **D**). Tubulin serves as a loading control. (**F**) Representative Hi-C contact maps (10 kb resolution, 1 Mb separation) from G2-synchronized wild type cells, and WAPL-dTAG cells in the presence and absence of dTAG-7. (**G**) Western blot confirming the presence of RAD21 and Sororin on chromatin in G2-synchronized WAPL-depleted (ΔWAPL and WAPL-dTAG) cells, as shown in (**B**, **D**). Histone H3 serves as a loading control. scsHi-C data from G2-synchronized WAPL-dTAG cells without target depletion (-dTAG-7) and upon WAPL depletion (ΔWAPL, +dTAG-7) are merged from *n* = 3 technical replicates and *n* = 2 independent experiments, respectively. Wild-type G2-synchronized scsHi-C data were reanalyzed from (Mitter et al, 2020; Data ref: Mitter et al, 2020a).

If loop extrusion promotes the accumulation of cohesive cohesin at high-level boundaries, then increasing the extrusion processivity should amplify this effect. To test this, we depleted WAPL in G2-synchronized cells (WAPL-dTAG) using a degradation tag system (Nabet et al, 2018) (Fig. EV5A–E), which increases cohesin residence time and promotes hyper-processive extrusion (Kueng et al, 2006; Gause et al, 2010; Tedeschi et al, 2013; Huis in ’t Veld et al, 2014; Wutz et al, 2017; Chan and Rubinstein, 2023; Gassler et al, 2017; Kiefer et al, 2023). While untreated WAPL-dTAG cells showed minor differences compared to wildtype (Fig. EV5E-F), we used the wild-type condition as a control for all comparisons to rule out any tag-related effects (Bondeson et al, 2022). Compared to published Hi-C data from wild-type G2-synchronized cells (Mitter et al, 2020; Data ref: Mitter et al, 2020a), Hi-C maps upon WAPL depletion showed stronger corner signals and increased long-range interactions (Fig. EV5F), consistent with enhanced extrusion, whereas chromatin-bound RAD21 and Sororin levels remained high (Fig. EV5G). ChIP-seq profiling showed that Sororin strongly concentrated at high-level TAD boundaries, reaching levels substantially higher than in wild type cells, while it decreased at low-level boundaries (Fig. 4A red tracks, C, upper panel). RAD21 displayed a similar redistribution (Fig. 4A, blue tracks, C, lower panel). Thus, hyper-processive extrusion enhances boundary stratification and amplifies the focal accumulation of both cohesive and loop-forming cohesin at high-level boundaries.

Together, these findings demonstrate that loop extrusion dynamics play a central role in the positioning of cohesive cohesin (Fig. 4D). Our data are consistent with a model in which loop-extruding cohesin can push cohesive cohesin toward boundary elements (Zhao et al, 2025), but further show that extrusion does not uniformly concentrate cohesive cohesin at all boundaries in interphase cells. Instead, loop extrusion can either increase or decrease cohesive cohesin enrichment at boundaries, depending on the interplay between boundary hierarchy and extrusion processivity. Extrusion dynamics thus provide a mechanism for distributing cohesive cohesin within the nested TAD architecture.

### Loop extrusion shapes cohesion zones

Because loop-extrusion processivity affects the positioning of both cohesin pools, we next asked how altering extrusion dynamics affects the nested architecture of linked and split loops in replicated chromosomes. To directly relate cohesin dynamics to chromosome conformation, we used scsHi-C to quantify both cis sister loops and trans sister contacts in G2 cells under conditions in which loop extrusion is either suppressed by NIPBL depletion or rendered hyper-processive by WAPL depletion.

**Figure 5:**
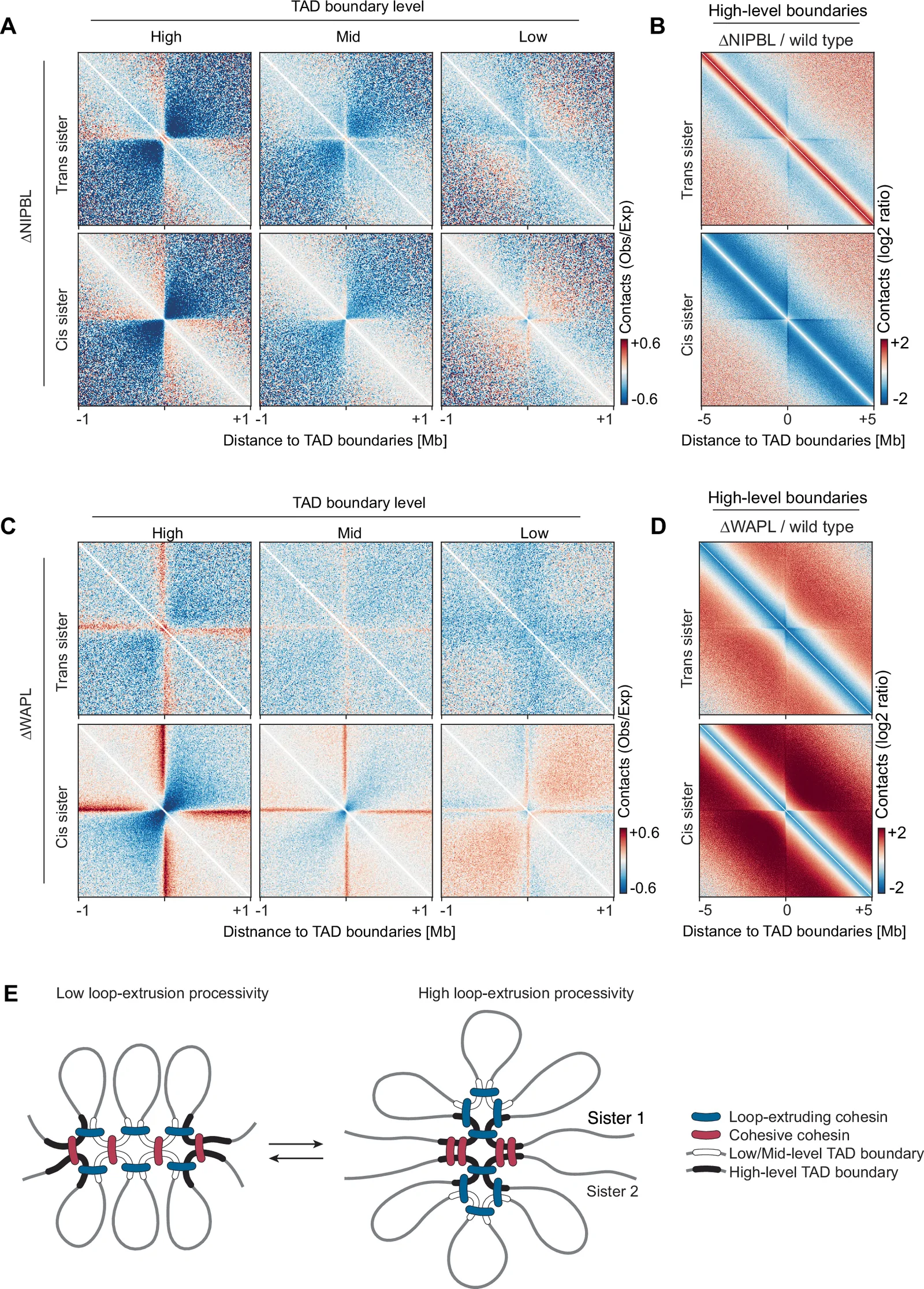
Loop extrusion positions trans sister contacts at high-level boundaries. (**A**) Average maps of trans sister (upper row) and cis sister (lower row) scsHi-C contacts (10 kb resolution) at ±1 Mb regions centered at high-, mid-, and low-level boundaries from G2-synchronized cells upon NIPBL depletion (ΔNIPBL and NIPBL-AID), normalized to genome-wide expected contact frequencies. (**B**) Ratio pileups (ΔNIPBL/wildtype) of trans sister (upper panel) and cis sister (lower panel) scsHi-C contacts (20 kb resolution) at ±5 Mb regions centered at high-level boundaries. Ratios represent contacts from NIPBL-depleted conditions (ΔNIPBL) normalized over wild-type conditions. (**C**) Average pileups of trans sister (upper row) and cis sister (lower row) scsHi-C contacts (10 kb resolution) at ±1 Mb regions centered at high-, mid-, and low-level boundaries from G2-synchronized cells upon WAPL depletion (ΔWAPL and WAPL-dTAG), normalized to genome-wide expected contact frequencies. (**D**) Ratio pileups (ΔWAPL/wild type) of trans sister (upper panel) and cis sister (lower panel) scsHi-C contacts (20 kb resolution) at ±5 Mb regions centered at high-level boundaries. Ratios represent contacts from WAPL-depleted conditions (ΔWAPL) normalized over wild-type conditions. (**E**) Model illustrating the role of loop extrusion processivity in positioning cohesive cohesin within the TAD hierarchy. scsHi-C data for ΔWAPL are merged from *n* = 2 independent experiments. Wild-type G2-synchronized scsHi-C data were reanalyzed from (Mitter et al, 2020; Data ref: Mitter et al, 2020a). ΔNIPBL G2-synchronized scsHi-C data reanalyzed from (Batty et al, 2023; Data ref: Batty et al, 2023).

To determine how loop extrusion contributes to the formation of cohesion zones, we compared published scsHi-C maps from G2-synchronized wild-type cells (Mitter et al, 2020; Data ref: Mitter et al, 2020a) with those from cells in which NIPBL was depleted after DNA replication (NIPBL-AID) (Batty et al*,* 2023; Data ref: Batty et al, 2023). The prominent contact stripes emanating from high-level boundaries in both cis and trans sister contact maps were almost completely lost (Fig. 5A). Contact-density ratios relative to wild type within a larger genomic window around high-level boundaries confirmed a marked depletion of boundary-centered enrichment and a redistribution of trans sister contacts toward shorter genomic distances (Fig. 5B). Thus, consistent with the dispersal of cohesive cohesin observed by ChIP-seq in NIPBL-depleted cells, inhibition of loop extrusion abolishes the formation of defined cohesion zones at high-level TAD boundaries.

We next asked how hyper-processive loop extrusion reshapes boundary-associated organization. We performed scsHi-C in WAPL-depleted G2 cells (WAPL-dTAG) and observed a pronounced reorganization of boundary features. In cis, contact stripes remained prominent, but insulation between adjacent domains was reduced (Fig. 5C, lower row), consistent with previous observations (Wutz et al, 2017; Haarhuis et al, 2017; Luppino et al, 2020). In trans, stripes at high-level boundaries remained visible but did not increase in strength compared to wild-type cells (Fig. 5C, upper row), despite elevated levels of Sororin-bound cohesin (Fig. 4A,C). Instead, contact-density ratios relative to wild type within a larger genomic window around high-level boundaries revealed a genome-wide shift toward longer-range trans sister contacts at boundaries and in adjacent regions (Fig. 5D), consistent with increased separation and reduced local alignment of sister chromatids. Together, these patterns suggest that under hyper-processive extrusion, high-level boundaries interact with a broader, less focused region on the sister chromatid.

Together, these perturbations show that loop extrusion dynamics fine-tune the nested architecture of replicated chromosomes (Fig. 5E). When extrusion is inhibited, trans sister interactions are no longer focused at high-level boundaries; when extrusion is hyper-processive, cis loops expand, and trans sister contacts extend to longer genomic distances. These results support a model in which balanced extrusion dynamics promote nested cis loop structures that form independently on each sister chromatid, while global sister chromatid alignment is maintained through discrete cohesion zones at high-level TAD boundaries.

### Clusters of CTCF sites define broad cohesion zones

**Figure EV6:**
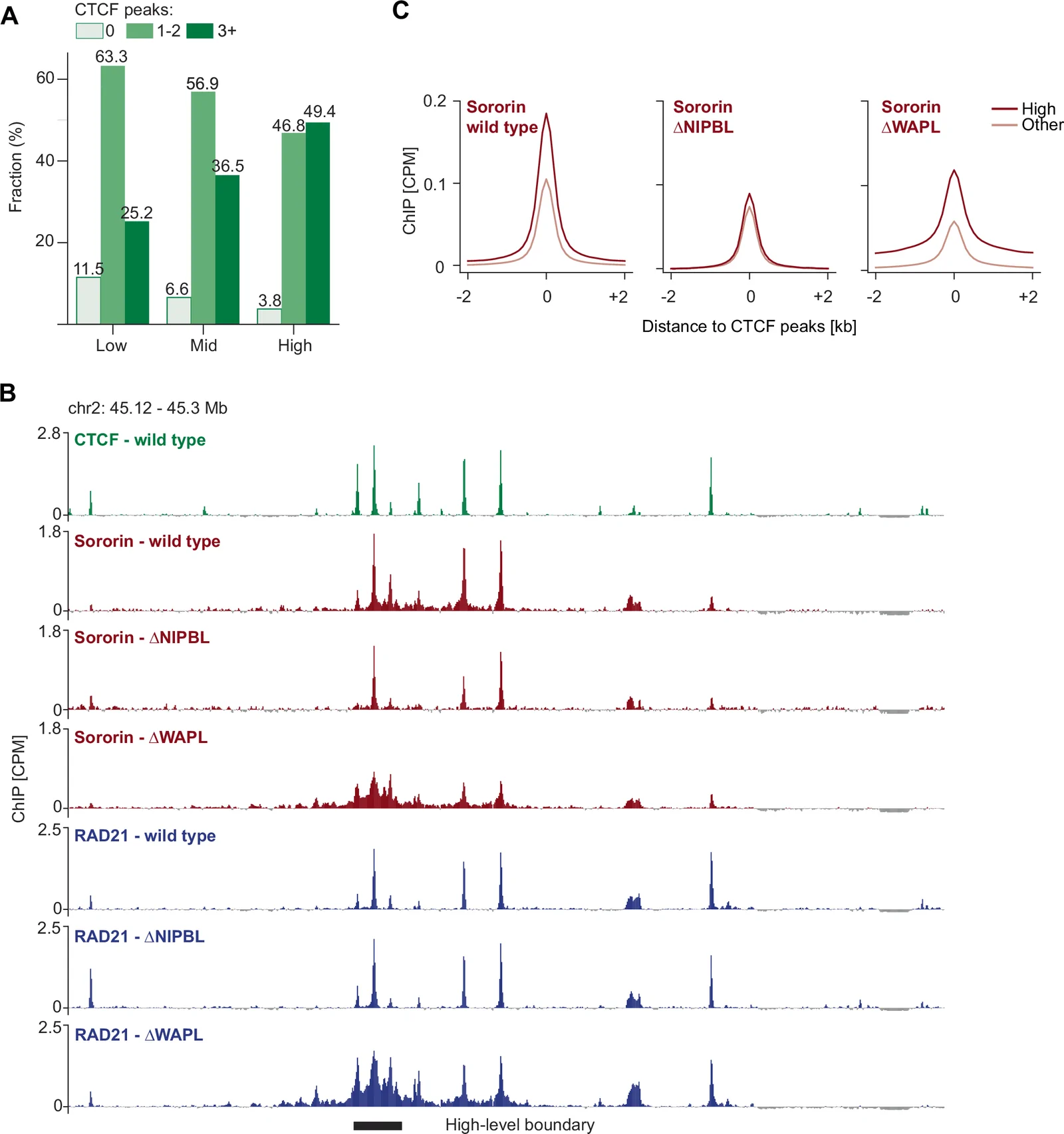
Characterization of CTCF clusters at high-level boundaries. (**A**) Fractions of low-, mid- and high-level boundaries containing 0, 1, 2, or ≥3 CTCF peaks. (**B**) Representative ChIP-Seq tracks (200 bp resolution) of CTCF (green) from G2-synchronized wildtype, and Sororin (red) and RAD21 (blue) from G2-synchronized wild type, NIPBL-depleted (ΔNIPBL, NIPBL-AID) and WAPL-depleted (ΔWAPL, WAPL-dTAG) HeLa cells. Position of a high-level boundary (10 kb) is indicated below. (**C**) Average profile of Sororin ChIP-Seq signal (100 bp resolution) in G2-synchronized cells under wild type (left panel), NIPBL-depleted (ΔNIPBL, middle panel) and WAPL-depleted (ΔWAPL, right panel) conditions at ±2 kb regions centered on isolated CTCF peaks (no additional CTCF peak within ±2.5 kb) and stratified by location: high-level boundaries (High) (*n* = 5989 peaks) or elsewhere in the genome (Other) (*n* = 44,600 peaks). ChIP-seq data were CPM-normalized, centered by subtracting the genome-wide mean and represent merged data from *n* = 3 (CTCF) or *n* = 2 (Sororin, RAD21) independent experiments.

High-level boundaries insulate stronger both in cis and trans (Fig. 3E), and strong TAD boundaries often contain multiple closely spaced CTCF sites (Kentepozidou et al, 2020; Chang et al, 2023; Anania et al, 2022). Consistent with this, we found that 49% of the high-level TAD boundaries contain three or more CTCF peaks (Fig. EV6A), in contrast to 36 and 25% of mid- and low-level boundaries, respectively. This raised the question of how cohesive cohesin is positioned relative to internal boundary organization. To address this, we analyzed the distribution of RAD21 and Sororin around CTCF sites located within high-level TAD boundaries compared with CTCF sites at other genomic regions.

**Figure 6:**
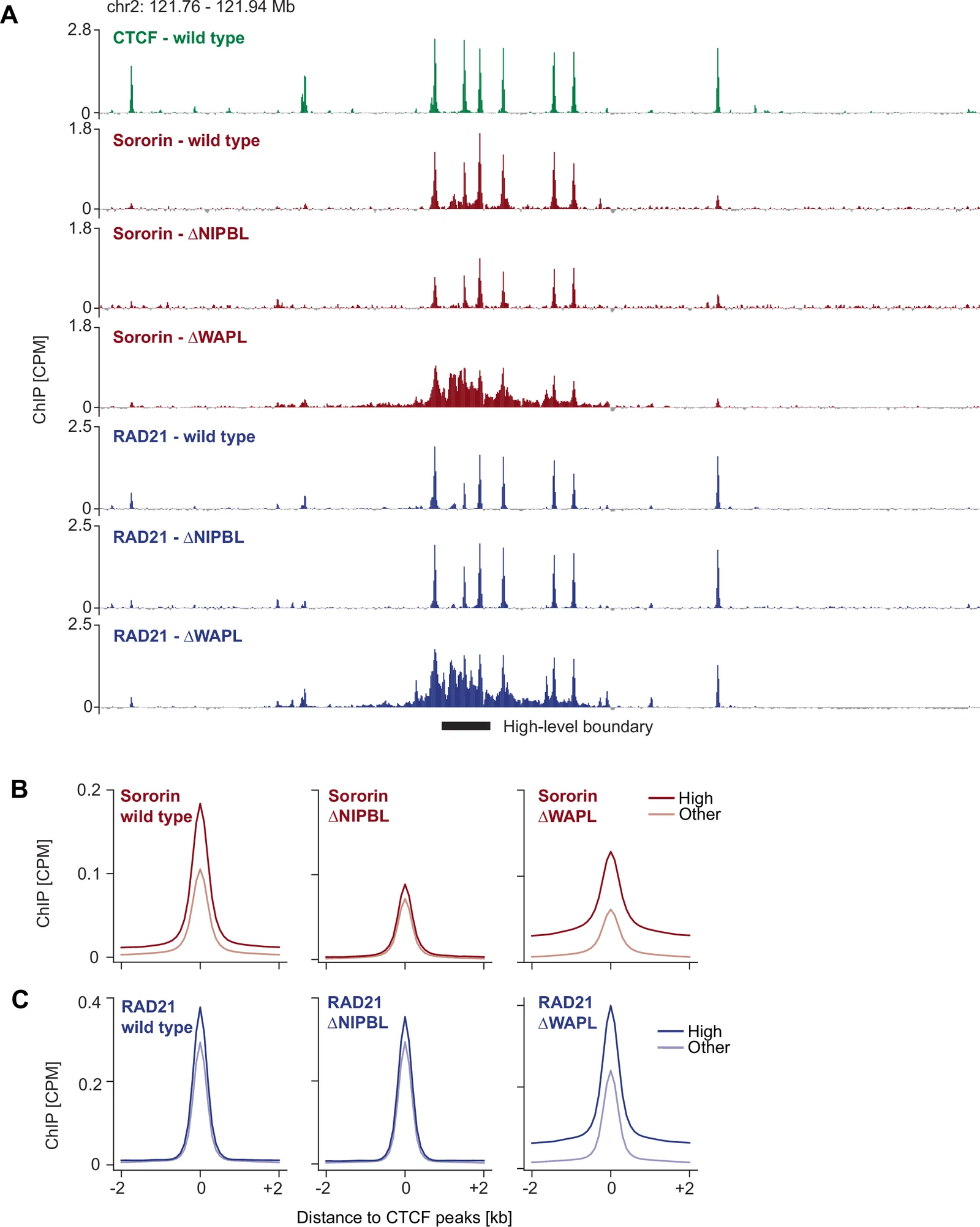
Clusters of CTCF sites define broad cohesion zones. (**A**) Representative ChIP-Seq tracks (200 bp resolution) of CTCF (green) from G2-synchronized wild type, and Sororin (red) and RAD21 (blue) from G2-synchronized wild type, NIPBL-depleted (ΔNIPBL, NIPBL-AID) and WAPL-depleted (ΔWAPL, WAPL-dTAG) cells. Position of a high-level boundary (10 kb long) is indicated below. (**B**) Average profile of Sororin ChIP-Seq signal (100 bp resolution) from G2 cells under wild type (left panel), ΔNIPBL (middle panel) and ΔWAPL (right panel) conditions at ±2 kb regions centered at CTCF peaks within high-level boundaries (High) (*n* = 8034 peaks) or elsewhere in the genome (Other) (*n* = 53,351 peaks). (**C**) Average profile of RAD21 ChIP-Seq signal (100 bp resolution) from G2 cells under wild type (left panel), ΔNIPBL (middle panel) and ΔWAPL (right panel) conditions at ±2 kb regions window centered at CTCF peaks within high-level boundaries (High) (*n* = 8034 peaks) or elsewhere in the genome (Other) (*n* = 53,351 peaks). ChIP-seq data were CPM-normalized, centered by subtracting the genome-wide mean and represent merged data from *n *= 3 (CTCF) or *n* = 2 (Sororin, RAD21) independent experiments.

Inspection of representative loci showed that in G2-synchronized wild-type cells, Sororin, like RAD21, formed sharp peaks centered on CTCF peaks (Figs. 6A and EV6B). Within high-level boundary regions, however, Sororin signal remained elevated between neighboring CTCF sites compared to the genome-wide average. This suggests that cohesive cohesin is retained not only at individual CTCF sites but also across the intervening boundary region.

To test whether this pattern is a general feature of high-level boundaries, we performed aggregate analyses across all CTCF peaks. At CTCF peaks within high-level boundary regions of G2-synchronized wild-type cells, Sororin displayed both increased peak amplitude and an elevated inter-peak baseline compared with CTCF peaks elsewhere in the genome (Fig. 6B, left panel), whereas RAD21 showed only a modest elevation of inter-peak signal (Fig. 6C, left panel). To assess whether the elevated baseline could be explained by contributions from neighboring CTCF peaks, we analyzed Sororin occupancy at CTCF peaks without other peaks in their close vicinity (±2.5 kb). Baseline Sororin signal remained elevated at high-level boundaries compared to elsewhere in the genome (Fig. EV6C), indicating that the pronounced inter-peak enrichment of Sororin is not only attributable to signal from adjacent CTCF peaks. Thus, high-level TAD boundary regions form extended cohesion zones in which cohesive cohesin accumulates both at CTCF peaks and across the intervening boundary interval.

Since loop extrusion positions cohesive cohesin to boundaries, it may also regulate the fine-scale distribution of cohesive cohesin around CTCF sites. When we analyzed Sororin binding in G2-synchronized NIPBL-depleted cells (NIPBL-AID), the Sororin signal was markedly reduced at CTCF peaks within boundary regions, and the elevated inter-peak baseline observed in wild-type cells almost completely disappeared (Fig. 6A, red tracks, B, middle panel). Thus, loop extrusion is required to concentrate cohesive cohesin not only at CTCF sites within TAD boundaries but also across the intervening regions that define extended cohesion zones.

To assess how hyper-processive loop extrusion reshapes the fine-scale boundary organization, we examined the effects of WAPL depletion in G2-synchronized cells (WAPL-dTAG) on cohesin distribution. ChIP-seq profiles revealed a striking redistribution of Sororin within high-level TAD boundaries: Sororin signal at CTCF peaks decreased relative to the genome-wide average, both within boundary regions and across the genome, while the inter-peak baseline within boundary regions became strongly elevated (Fig. 6A, red tracks, B, right panel). RAD21 showed a similar accumulation between neighboring CTCF sites at high-level boundaries (Fig. 6A, blue tracks, C, right panel). These results indicate that hyper-processive extrusion reshapes cohesion zones by shifting both cohesin pools from sharp, CTCF-centered peaks into broader inter-peak regions within high-level TAD boundaries.

Together, these results show that clustering of CTCF sites at high-level boundaries contributes to the establishment of broad cohesion zones, where loop extrusion shapes the fine-scale distribution of cohesive cohesin: under wild-type conditions, Sororin is enriched both at CTCF peaks and across the intervening boundary interval, whereas hyper-processive extrusion shifts Sororin from individual peaks into the inter-peak regions. Thus, loop extrusion dynamics actively distribute cohesive cohesin across CTCF clusters, transforming discrete binding sites into continuous cohesion zones.

## Discussion

The replicated interphase genome faces a fundamental organizational challenge: it must keep sister chromatids in proximity to enable homology-directed DNA repair and faithful chromosome segregation while maintaining the dynamic loop structures that support gene regulation. Both requirements are mediated by cohesin, yet cohesin’s two principal activities are inherently antagonistic: loop extrusion promotes chromatid separation, whereas cohesion resists separation and can impede loop extrusion (Goloborodko et al, 2016; Mitter et al, 2020; Chatzidaki et al, 2021; Batty et al, 2023; Bastié et al, 2024; Zhao et al, 2025; Ochs and Gerlich, 2026). Our findings reconcile this paradox by revealing a spatial partitioning of cohesin function on replicated chromosomes. Rather than co-localizing uniformly with loop-extruding cohesin, cohesive cohesin concentrates at discrete genomic positions defined by the nested TAD hierarchy. This hierarchy relies on active loop extrusion to continually sweep cohesive cohesin from intervening domains towards the strongest barriers. Such segmentation creates cohesion zones that maintain large-scale sister chromatid alignment, while loop extrusion predominates within the intervening chromatin. Thus, hierarchical boundary organization enables mechanical pairing and dynamic folding to coexist on the same replicated chromosome template.

### Interplay of cohesin and CTCF defines cohesion zones

Our data are consistent with a model in which loop extrusion moves cohesive cohesin toward boundary elements, as proposed from studies in mitotic cells (Zhao et al, 2025). In interphase, however, the two cohesin pools diverge in their distributions, raising the question of how cohesive cohesin becomes selectively enriched at high-level boundaries. This divergence may reflect dynamic interactions between cohesin and CTCF. Individual CTCF peaks act as semi-permeable barriers that transiently stall cohesin yet can be bypassed (Fudenberg et al, 2016; Nuebler et al, 2018; Davidson et al, 2023; preprint: Rudnizky et al, 2025). In contrast, high-level boundary regions containing multiple CTCF peaks establish more effective barriers, maintaining insulation even upon occasional bypass of individual CTCF peaks (Kentepozidou et al, 2020; Chang et al, 2023; Chang and Noordermeer, 2024). In this context, extrusion can enrich cohesin at boundary regions but can also redistribute cohesin away from individual CTCF peaks, depending on extrusion dynamics and local boundary context. Loop extrusion processivity then dictates the degree to which cohesive cohesin is biased toward strong boundaries versus weak ones. Notably, loop-extruding cohesin dissociates within minutes and reforms loops elsewhere in the genome, whereas cohesive cohesin persists on chromatin for many hours throughout interphase (Gerlich et al, 2006b; Ladurner et al, 2016; Holzmann et al, 2019). This distinct chromatin binding time provides a simple mechanism for selective accumulation: cohesive cohesin accumulates at high-level boundaries over time, whereas the loop-extruding pool remains more evenly distributed across boundaries of different hierarchy levels. Barrier permeability, loop extrusion processivity, and differential cohesin turnover on chromatin thereby give rise to distinct distributions of loops and cohesive linkages within the same CTCF-defined positional framework.

### A shared 3D architecture for gene regulation and DNA repair

The formation of cohesion zones on replicated chromosomes provides a structural framework for homology-directed DNA repair. By limiting the genomic range of trans sister contacts, cohesion zones confine homology search to regions on the order of a high-level TAD. Such spatially focused sampling offers a major kinetic advantage for identifying homologous repair templates compared with a genome-wide search (Teloni et al, 2025; preprint: Yeh et al, 2025; Marin-Gonzalez et al, 2025; Renkawitz et al, 2014; Haber, 2018; Ochs and Gerlich, 2026). At the same time, the sparseness of cohesion zones along chromosome arms suggests why DNA double-strand breaks trigger local architectural remodeling to further enhance search efficiency, by increasing local looping and by forming a cohesive clamp near the break (Arnould et al, 2021; Piazza et al, 2021; Marin-Gonzalez et al, 2025; Teloni et al, 2025). Cohesin thereby effectively converts a high-level TAD into a homology-sampling unit, enabling rapid and accurate repair.

Restricting cohesion to defined zones may also minimize the extent to which cohesive cohesin impedes loop extrusion (Chatzidaki et al, 2021; Bastié et al, 2024; Zhao et al, 2025), leaving most of the chromatin fiber permissive for extrusion-driven looping that supports enhancer–promoter communication (Robson et al, 2019; Karpinska and Oudelaar, 2023; Buckley et al, 2025). The nested TAD hierarchy, therefore, separates functions across scales: high-level boundaries maintain sister alignment, whereas intervening domains maintain dynamic cis loop architectures on each chromatid. By integrating these distinct modes of cohesin activity within the TAD hierarchy, replicated chromosomes can support homology-directed DNA repair and transcriptional regulation within the same 3D architecture.

### Conclusions and future directions

Our findings raise important questions about the organization of replicated chromosomes. First, where and how is cohesion initially established during the S phase, and how do these sites relate to the steady-state accumulation of cohesive cohesin in G2? Second, which features, besides CTCF sites, define where cohesion zones form, including chromatin state, transcriptional context, and additional architectural factors? Third, to what extent is cohesive cohesin positioning influenced by forces other than cohesin-mediated loop-extrusion, for example by other SMC complexes such as SMC5/6 (Kim et al, 2023; Davidson and Peters, 2021; Hoencamp and Rowland, 2023), by replication-associated machineries such as MCM (Dequeker et al, 2022), or by the transcription machinery (Borrie et al, 2023, 2017; Lengronne et al, 2004; Banigan et al, 2023; Busslinger et al, 2017; Glynn et al, 2004; Ocampo-Hafalla et al, 2016)? Our sister-specific conformation capture, combined with protein localization and degron-based perturbations, provides a powerful framework to address these and other questions emerging from our study.

Our study highlights a general design principle by which SMC-regulated chromosome architectures can be tuned by balancing processivity, residence time, boundary strength, and topological linkage. In mitosis, condensin I and II generate nested loop architectures through differential processivity and residence time (Gerlich et al, 2006a; Gibcus et al, 2018; Walther et al, 2018; Dey et al, 2023), and the removal of CTCF-defined boundaries coincides with a major reconfiguration of chromosome folding (Naumova et al, 2013; Nagano et al, 2017; Oomen et al, 2019; Mitter et al, 2020). In developing B cells, reduced expression of WAPL increases cohesin loop-extrusion processivity to enable long-range V(D)J recombination (Hill et al, 2020; Peters, 2021; Zhang et al, 2022). In replicated chromosomes, we find that an interplay between cohesin pools with distinct kinetics establishes discrete cohesion zones that tether sister chromatids while preserving a dynamic landscape of regulatory loops. Together, these findings suggest that a single positional framework, encoded by the genomic positions of CTCF binding sites, can generate fundamentally different chromosome architectures across the cell cycle. By tuning extrusion dynamics, boundary strength, and topological linkage, this framework supports diverse functions such as transcription, DNA repair, and chromosome segregation.

## Methods

**Table:** Reagents and tools table

Reagent/resource	Reference or source	Identifier or catalog number
**Experimental models**		
HeLa Kyoto wildtype	S. Narumiya, Kyoto University, Japan	Internal ID 1
HeLa Kyoto mEGFP-AID2-NIPBL (NIPBL-AID)	Batty et al, 2023	Internal ID 2045
HeLa Kyoto HA-FKBP12^F36V^-WAPL (WAPL-dTAG)	Batty et al, 2023	Internal ID 2096
CTCF-EGFP-AID1 (CTCF-AID)	Wutz et al, 2017	Internal ID 1900
CTCF-AID1-mKate2_SCC1-HALO (CTCF-AID/SCC1-HALO)	Wutz et al, 2025	Internal ID 1901
**Recombinant DNA**		
**Antibodies**		
Rabbit anti-CTCF	Cell Signaling Technology	Cat #2899
Rabbit anti-GAPDH	Abcam	Cat #ab9485
Mouse anti-phospho-H3 (Ser10)	Millipore	Cat #05-806
Rabbit anti-H3	Cell Signaling Technology	Cat #4499
Rabbit anti-SCC4/MAU2	Abcam	Cat #ab183033
Rat anti-NIPBL	Absea	Cat #010702F01
Rabbit anti-RAD21 (ChIP)	Abcam	Cat #ab992
Rabbit anti-RAD21 (Immunoblotting)	Abcam	Cat #ab217678
Mouse anti-Sororin clone 3D5-G10 (Immunoblotting)	Max Perutz Labs Monoclonal Antibody Facility, Batty et al, 2023	internal ID 432
Rabbit anti-Sororin (ChIP)	Abcam	Cat #192237
Rabbit anti-Tubulin	Abcam	Cat #52866
Rabbit anti-Vinculin	Abcam	Cat #129002
Mouse anti-WAPL	Santa-Cruz Biotechnology	Cat #sc-365189
Goat anti-mouse-Alexa Fluor 488	Molecular Probes	Cat #A11001
Goat anti-mouse-HRP	Bio-Rad	Cat #1706516
Goat anti-rabbit-HRP	Bio-Rad	Cat #1706515
Goat anti-rat-HRP	Amersham	Cat #NA935
**Oligonucleotides and other sequence-based reagents**		
**Chemicals, enzymes and other reagents**		
Affi-Prep Protein A beads	Bio-Rad	Cat #1560005
AMPure XP beads	Beckman Coulter	Cat #A63881
Aphidicolin	Sigma-Aldrich	Cat #A0781
Benzonase	Sigma-Aldrich	Cat #70746
Biotin-14-dATP	Invitrogen	Cat #19524016
Blasticidin S	Thermo Fisher Scientific	Cat #A1113903
Bovine serum albumin (BSA)	VWR Chemicals	Cat #422351S
Clarity Max ECL substrate	Bio-Rad	Cat #1705062
cOmplete Mini protease inhibitor cocktail	Roche	Cat #11836170001
1,4-Dithiothreitol (DTT)	Millipore	Cat #1114740001
DMEM high glucose	Capricorn Scientific	Cat #DMEM-HPA-P10
DpnII	New England Biolabs	Cat #R0543
10X DpnII digestion buffer	New England Biolabs	Cat #B0543
Dynabeads^TM^ MyOne^TM^ Streptavidin C1 beads	Invitrogen	Cat #65001
Ethanol	Sigma-Aldrich	Cat #32221
Fetal bovine serum (FBS)	Gibco	Cat #10270-106
Formaldehyde 16%	Thermo Fisher Scientific	Cat #28906
Formamide	Sigma-Aldrich	Cat #F9037
GlutaMAX	Invitrogen	Cat #35050038
Indole-3-acetic acid (IAA)	Sigma-Aldrich	Cat #I5148
Klenow polymerase	New England Biolabs	Cat #M0210
Milk powder	Maresi	
NEB2 Buffer	New England Biolabs	Cat #B7002
Nonidet P 40 Substitute (NP-40)	Sigma-Aldrich	Cat #74385
Osmium tetroxide (OsO_4_)	Sigma-Aldrich	Cat #75632
Phenylmethanesulfonyl fluoride	Sigma-Aldrich	Cat #93482
5-Phenyl-indole-3-acetic acid (5-Ph-IAA)	Bio Academia	Cat #30-003
Penicillin-Streptomycin	Sigma-Aldrich	Cat #P0781
Propidium iodide	Sigma-Aldrich	Cat #81845
Proteinase K	Qiagen	Cat #19131
RNAse A	Qiagen	Cat #19101
RO-3306	Sigma-Aldrich	Cat #SML0569
Sodium deoxycholate	Sigma-Aldrich	Cat #30970
Sodium dodecyl sulfate (SDS)	Serva	Cat #20765.02
dTAG-7	Tocris	Cat #6912
4-thio-thymidine (4sT)	Carbosynth	Cat #NT06341
Thymidine	Sigma-Aldrich	Cat #T1895
Trypsin		
Triton X-100	Sigma-Aldrich	Cat #327371000
Tween-20	Sigma-Aldrich	Cat #P1379
T4 DNA ligase	Thermo Fisher Scientific	Cat #EL0011
10X T4 DNA ligase buffer	Thermo Fisher Scientific	Cat #B69
		Cat #
		Cat #
		Cat #
**Software**		
FlowJo v.10	https://flowjo.com	
NovoExpress	Agilent, https://www.agilent.com/en/product/research-flow-cytometry/flow-cytometry-software/novocyte-novoexpress-software-1320805	
Nextflow	Ewels et al, 2020	
nf-core/chipseq	Patel et al, 2020	v1.2.1
SAMtools	Li et al, 2009	v1.15
deepTools	Ramírez et al, 2016	v3.5.4
MACS2	Zhang et al, 2008	v2.2.9.1
BEDTools	Quinlan and Hall, 2010	v2.31.1
pybbi	Abdennur, 2025	v0.4.2
NeoLoopFinder	Wang et al, 2021	v0.4.3
scsHi-C pipeline	Langer, 2021	tag 2b09aa1
pairtools	Open2C, 2023	v1.1.3
cooler	Abdennur, 2025	v0.10.2
OnTAD	An et al, 2019	v1.2
cooltools	Open2C, 2024a	v0.7.1
Python	www.python.org	v3.10
NumPy	Harris et al, 2020	v1.25.2
Pandas	The pandas development team, 2023	v1.5.3
SciPy	Virtanen et al, 2020	v1.15.2
bioframe	Open2C, 2024b	v0.8.0
pybigtools	Huey and Abdennur, 2024	v0.2.4
HiCognition	Langer et al, 2023	
IGV	Robinson et al, 2011	
HiGlass	Kerpedjiev et al, 2018	
Matplotlib	Hunter, 2007	v3.9.4
Seaborn	Waskom, 2021	v0.13.2
JupyterLab	www.jupyter.org	
**Other**		
NuPAGE Bis-Tris gel, 4–12%	Thermo Fisher Scientific	Cat #NP0335BOX
NuPAGE Tris-Acetate gel, 3–8%	Thermo Fisher Scientific	Cat #EA0378BOX
Amersham Hybond P Western blotting membrane, PVDF	Sigma-Aldrich	GE10600023
DNeasy Blood & Tissue Kit	Qiagen	Cat #69506
Monarch PCR & DNA cleanup kit	New England Biolabs	T1030
NEBNext Ultra II DNA Library Prep Kit	New England Biolabs	E7645
Covaris S2 sonicator	Covaris	
Bioruptor	Diagenode	
NovoCyte Penteon Flow Cytometer	Agilent	
ChemiDoc Imaging System	Bio-Rad	
Illumina NextSeq2000	Illumina	
Illumina NextSeq 550	Illumina	
Illumina NovaSeq SP	Illumina	
Illumina NovaSeqX	Illumina	

### Cell culture

HeLa cells (Kyoto strain) were provided by S. Narumiya (Kyoto University) and validated via Multiplex Human Cell line Authentication (MCA). Cells were maintained in high-glucose DMEM (Capricorn Scientific, DMEM-HPA-P10) supplemented with 10% (v/v) FBS (Gibco, 10270-106), 1% (v/v) GlutaMAX (Invitrogen, 35050038), and 1% (v/v) penicillin-streptomycin (Sigma-Aldrich, P0781), hereafter referred to as “growth medium”. NIPBL-AID and WAPL-dTAG lines were maintained under selection with 6 μg/ml blasticidin S (Thermo Fisher Scientific, A1113903). All cultures were grown at 37 °C in a humidified incubator with 5% CO_2_ and routinely tested for mycoplasma.

### Cell synchronization and targeted protein degradation

For synchronization at the G1/S boundary, asynchronous cells were treated with 2 mM thymidine (Sigma-Aldrich, T1895) for 18 h, washed twice with prewarmed growth medium, and released for 8 h. Cells were then re-arrested with 3 μg/ml aphidicolin (Sigma-Aldrich, A0781) for 16 h. G1/S populations were harvested immediately following this block. For synchronization in G2 phase, cells were released from aphidicolin into fresh growth medium for 4 h and subsequently treated with 9 μM RO-3306 (Sigma-Aldrich, SML0569) for 20 h prior to harvest.

To induce targeted protein depletion, degron ligands were added to the medium as follows:

- NIPBL-AID: 1 μM 5-phenyl-indole-3-acetic acid (5-Ph-IAA; Bio Academia, 30-003) for 16 h, starting 8 h post-aphidicolin release.
- WAPL-dTAG: 1 μM dTAG-7 (Tocris, 6912) for 16 h, starting 8 h post-aphidicolin release.
- CTCF-AID/SCC1-HALO (scsHi-C): 500 μM indole-3-acetic acid (IAA; Sigma-Aldrich, I5148) for 12 h, starting 12 h post-aphidicolin release.
- CTCF-AID (ChIP-Seq/Chromatin fractionation): 500 μM IAA for 16 h, starting 8 h post-aphidicolin release.

For sister-chromatid-sensitive Hi-C (scsHi-C) labeling, 2 mM 4-thiothymidine (4sT; Carbosynth, NT06341) was added at the onset of the aphidicolin block and maintained until harvest.

### Flow cytometry and cell cycle analysis

Cell cycle distribution was determined by quantifying DNA content via propidium iodide (PI) staining and Histone H3 Serine 10 phosphorylation (pH3S10). Unless otherwise stated, all centrifugation steps were performed at 1100 × *g* for 1 min. Harvested cells (2 × 10^5^) were washed with PBS and fixed in 70% ethanol (Sigma-Aldrich, 32221) for at least 1 h at 4 °C. Fixed cells were permeabilized with 0.25% Triton X-100 (Sigma-Aldrich, 327371000) in PBS for 15 min on ice and subsequently incubated with 0.25 μg anti-phospho-H3 (Ser10) antibody (Millipore, 05-806) for 1 h at room temperature (RT). After washing with 1% bovine serum albumin (BSA; VWR Chemicals, 422351S) in PBS, cells were incubated with Alexa Fluor 488-conjugated anti-mouse IgG (1:300; Molecular Probes, A11001) for 30 min at RT. Cells were washed and resuspended in PBS containing 50 μg/ml propidium iodide (PI) (Sigma-Aldrich, 81845) and 200 μg/ml RNAse A (Qiagen, 19101) for 30 min at RT. Data were acquired on a Penteon flow cytometer using NovoExpress software. The PI signal was detected using a B615 (615/20) filter, and Alexa Fluor 488 was detected using a B525 (525/45) filter. Data analysis was performed using FlowJo v.10. The gating strategy consisted of: (1) identification of the main cell population using FSC-A/SSC-A, (2) doublet exclusion using FSC-A/FSC-H and PI-A/PI-H, (3) quantification of cell cycle phases by plotting pH3S10 intensity (y-axis) against DNA content (x-axis).

### Chromatin fractionation

Synchronized cells (up to 2 × 10^6^) were harvested by trypsinization in the presence of 9 μM RO-3306 (Sigma-Aldrich, SML0569) and appropriate protein degron ligands to maintain cell cycle state and protein depletion. Cells were washed in PBS, collected by centrifugation (2000 × *g*, 3 min, 4 °C), and stored at −70 °C. Pellets were resuspended in ice-cold lysis buffer (20 mM Tris-HCl, pH 7.5, 100 mM NaCl, 5 mM MgCl_2_, 10% glycerol, 0.2% NP-40 (Sigma-Aldrich, 74385), 1 mM 1,4-dithiotreitol (DTT) (Millipore, 1114740001) and 1X cOmplete Mini protease inhibitor cocktail (Roche, 11836170001)) and incubated for 20 min on ice. The soluble fraction was collected following centrifugation (2000 × *g*, 3 min, 4 °C). The remaining chromatin pellet was washed three times with ice-cold lysis buffer and then solubilized by incubation with Benzonase (1:500; Sigma-Aldrich, 70746) in lysis buffer for 30 min at room temperature. The remaining insoluble fraction was removed by centrifugation (16,000 × *g*, 20 min, 4 °C), and the supernatant was collected as the chromatin-bound fraction.

### Western blotting

Protein samples were resolved on NuPAGE 4–12% Bis-Tris (Thermo Fisher Scientific, NP0335BOX) or, specifically for NIPBL detection, 3–8% Tris-Acetate (Thermo Fisher Scientific, EA0378BOX) polyacrylamide gels. Proteins were transferred to 0.45 µm PVDF membranes (Amersham Hybond P; Sigma-Aldrich, GE10600023) in transfer buffer containing 20% ethanol, or without ethanol for NIPBL detection. Membranes were blocked in 5% non-fat milk in PBS containing 0.05% Tween-20 (Sigma-Aldrich, P1379) (PBST) and incubated overnight at 4 °C with the following primary antibodies: anti-CTCF (1:1000, Cell Signaling Technology, 2899), anti-MAU2 (1:1000, Abcam, ab183033), anti-NIPBL (1:800, Absea, 010702F01), anti-WAPL (1:1000, Santa-Cruz Biotechnology, sc-365189), anti-RAD21 (1:1000, Abcam, ab992 or ab217678), and anti-Sororin (1:50, in house; as described in (Batty et al, 2023)). Loading controls were detected using antibodies against GAPDH (1:2500, Abcam, ab9485), α-Tubulin (1:1000, Abcam, ab52866), Vinculin (1:10,000, Abcam, ab129002), and Histone H3 (1:1000, Cell Signaling Technology, 4499). Membranes were washed three times in PBS for 10 min each and subsequently incubated with HRP-conjugated secondary antibodies: goat anti-rabbit (1:5000, Bio-Rad, 1706515), goat anti-mouse (1:5000, Bio-Rad, 1706516) and goat anti-rat (1:5000, Amersham, NA935). Membranes were washed three times in PBS for 10 min each. Chemiluminescent signals were developed using Clarity Max ECL substrate (Bio-Rad, 1705062) and visualized on a ChemiDoc imaging system (Bio-Rad).

### Hi-C and scsHi-C sample preparation

Synchronized cells (2 × 10^6^) were harvested by trypsinization in the presence of appropriate compounds to maintain cell cycle phase and protein depletion. Cells were washed with PBS, fixed in 1% formaldehyde (Thermo Fisher Scientific, 28906) for 4 min at room temperature (RT) and quenched with 20 mM Tris (pH 7.5). Pellets were collected and stored at −70 °C. Unless otherwise stated, all centrifugation steps were performed at 2500 × *g* for 5 min. Cells were incubated in ice-cold lysis buffer (10 mM Tris-HCl, pH 8.0, 10 mM NaCl, 0.2% NP-40 (Sigma-Aldrich, 74385), 1x cOmplete Mini protease inhibitor cocktail (Roche, 11836170001)) for 30 min at 4 °C. Following lysis, nuclei were collected by centrifugation and washed once with 1x ice-cold DpnII digestion buffer (NEB, B0543). Nuclei were permeabilized in digestion buffer containing 0.1% SDS (Serva, 20765.02) for 10 min at 65 °C with shaking (300 rpm). SDS was quenched by the addition of 1% Triton X (Sigma-Aldrich, 327371000) for 10 min on ice. Chromatin was digested overnight at 37 °C with 375 U DpnII (NEB, R0543) at 800 rpm, followed by heat inactivation at 65 °C for 20 min. DNA ends were biotinylated using 50 U Klenow polymerase (NEB, M0210) in 1x NEB2 Buffer (NEB, B7002) supplemented with 38 µM each of biotin-14-dATP (Invitrogen, 19524016), dCTP, dGTP and dTTP for 1 h at 37 °C (800 rpm). DNA fragments were ligated for 4 h at RT in a reaction containing 1x T4 DNA ligase buffer (Thermo Fisher Scientific, B69), 0.1% Triton X, 100 µg/ml BSA (VWR Chemicals, 422351S), and 50 U T4 DNA ligase (Thermo Fisher Scientific, EL0011). Crosslinks were reversed overnight at 65 °C (Hi-C) or for 6 h at 65 °C (scsHi-C). DNA was purified using the DNeasy Blood & Tissue Kit (Qiagen, 69506) and sheared to a target size of ~500 bp using a Covaris S2 sonicator (duration: 25 s, duty cycle: 10%, intensity: 5.0, cycles/burst: 200). Following size selection with AMPure XP beads (Beckman Coulter, A63881), biotinylated fragments were captured using Dynabeads^TM^ MyOne^TM^ Streptavidin C1 beads (Invitrogen, 65001) for 60 min at RT with rotation. For scsHi-C, an additional thiol-conversion step was performed using 0.45 mM osmium tetroxide (OsO_4_) (Sigma-Aldrich, 75632) and 180 mM NH_4_Cl (pH 8.88) for 3 h at 60 °C, followed by ethanol precipitation. Libraries were prepared using NEBNext Ultra II DNA Library Prep Kit (NEB, E7645). DNA was eluted from streptavidin beads using 95% formamide (Sigma-Aldrich, F9037) in 10 mM EDTA (pH 8.0) for 2 min at 65 °C. Following ethanol precipitation, libraries were amplified according to the NEBNext Ultra II protocol and purified with AMPure XP beads (0.55x ratio). Libraries were sequenced on Illumina NextSeq2000, NovaSeq SP or NovaSeqX platforms.

### ChIP-Seq

Synchronized cells (1 × 10^7^) were harvested by trypsinization in the presence of appropriate compounds to maintain cell cycle phase and protein depletion. Cells were washed with PBS, fixed in 1% formaldehyde (Thermo Fisher Scientific, 28906) for 10 min at room temperature (RT) and quenched with 125 mM glycine for 5 min at RT. Pellets were collected and stored at −70 °C. Unless otherwise stated, all centrifugation steps were performed at 1100×*g* for 1 min. Affi-prep Protein A beads (Bio-Rad, 1560005) were blocked overnight at 4 °C in blocking buffer (20 mM Tris-HCl, pH 8.0, 2 mM EDTA, pH 8.0, 1% Triton X (Sigma-Aldrich, 327371000), 150 mM NaCl, 1 mM phenylmethanesulfonyl fluoride (PMSF; Sigma-Aldrich, 93482), 0.1 mg/ml BSA (VWR Chemicals, 422351S)), washed and resuspended in dilution buffer (blocking buffer without BSA). Fixed cells were washed in ice-cold lysis buffer (50 mM Tris-HCl, pH 8.0, 10 mM EDTA pH 8.0, 1% SDS (Serva, 20765.02), 1 mM PMSF, onefold cOmplete Mini protease inhibitor cocktail (Roche, 11836170001)) and collected by centrifugation (1700×*g* for 2 min at 4 °C). Chromatin was fragmented to an average size of 200–500 bp using Diagenode Bioruptor (high intensity, 12 cycles, 30 s on/30 s off). After removing a 10 µl aliquot as input control, chromatin was diluted 1:10 in dilution buffer and pre-cleared with blocked Affi-Prep Protein A beads for 1 h at 4 °C. Pre-cleared chromatin was incubated overnight at 4 °C with the following antibodies: 0.3 µg CTCF (Cell Signaling Technology, 2899), 10 µg RAD21 (Abcam, ab992), and 10 µg Sororin (Abcam, ab192237). Chromatin-antibody complexes were captured with Affi-Prep Protein A beads for 3 h at 4 °C. Beads were washed twice with each of the following buffers: low-salt buffer (20 mM Tris-HCl, pH 8.0, 2 mM EDTA, pH 8.0, 1% Triton X, 150 mM NaCl, 0.1% SDS, and 1 mM PMSF), high salt buffer (20 mM Tris-HCl, pH 8.0, 2 mM EDTA, pH 8.0, 1% Triton X, 500 mM NaCl, 0.1% SDS, and 1 mM PMSF), LiCl buffer (10 mM Tris-HCl, pH 8.0, 2 mM EDTA, pH 8.0, 250 mM LiCl, 0.5% NP-40 (Sigma-Aldrich, 74385), 0.5% sodium deoxycholate (Sigma-Aldrich, 30970)), and TE buffer (10 mM Tris-HCl, pH 8.0, 1 mM EDTA, pH 8.0). Chromatin was eluted (25 mM Tris-HCl, pH 8.0, 5 mM EDTA, pH 8.0, 0.5% SDS) at 65 °C for 20 min with shaking (1200 rpm). ChIP and input samples were treated with RNAse A (100 mg/ml, Qiagen, 19101) and Proteinase K (20 mg/ml, Qiagen, 19131) for 1 h at 37 °C, followed by overnight incubation at 65 °C to reverse crosslinks. DNA was purified using the Monarch PCR & DNA Cleanup kit (NEB, T1030). Sequencing libraries were prepared using NEBNext Ultra II DNA Library Prep Kit (NEB E7645) and sequenced on Illumina NextSeq 550 or NovaSeqX platforms.

### ChIP-seq data processing

Sequencing reads were processed using the Nextflow (Di Tommaso et al, 2017) pipeline nf-core/chipseq v1.2.1 (Ewels et al, 2020; Patel et al, 2020). To ensure consistent read lengths across sequencing runs, reads were trimmed to 75 bp (--hard_trim5 75) and processed in single-end mode. Following read alignment to the human genome (hg19), replicate BAM files were merged using SAMtools v1.15 (Li et al, 2009). ChIP-seq signal tracks were generated in bigWig (Kent et al, 2010) format using deepTools v3.5.4 (Ramírez et al, 2016). For this step, mapped reads were extended to the fragment size estimated with MACS2 v2.2.9.1 (Zhang et al, 2008). Coverage was calculated in 50 bp genomic bins, excluding blacklisted regions (Amemiya et al, 2019) and duplicate reads, followed by normalization to counts per million (CPM). Downstream analyses utilized signal tracks from immunoprecipitated (IP) samples only.

### ChIP-seq peak calling

ChIP-seq peaks were called using MACS2 v2.2.9.1 (Zhang et al, 2008) with default parameters, utilizing immunoprecipitated (IP) samples as treatment and corresponding input samples as control. The effective genome size was set to 2,736,124,898 (hg19), corresponding to unique mappers with 75 bp read length. To define robust peaks set, calls overlapping blacklisted regions were removed using BEDTools v2.31.1 (Quinlan and Hall, 2010), and the remaining peaks were filtered by *q* value threshold of <1e-5. To ensure reproducibility, peaks were initially called on merged replicates; the final set retained only those merged peaks that overlapped with peaks called independently in at least two biological replicates.

### ChIP-seq average profiles

Average ChIP-seq profiles centered at regions of interest (TAD boundaries and CTCF peaks) were computed using pybbi v0.4.2 (Abdennur, 2025). For each region, ChIP-seq signals were extracted from bigWig files within defined genomic windows and aggregated into a fixed number of equally sized bins; specific window sizes and resolutions are indicated in the corresponding figure legends. To compensate for regional copy-number variation (CNV), the signal in each bin was divided by the corresponding copy number estimated from published Hi-C data of G2-synchronized HeLa cells (100 kb resolution) (Mitter et al, 2020; Data ref: Mitter et al, 2020a) using NeoLoopFinder v0.4.3 (Wang et al, 2021). Bin-wise signals of individual regions were averaged, and the genome-wide mean was subtracted from the averaged profiles to represent signal enrichment.

### ChIP-seq stackline profiles

To generate stackline profiles (heatmaps), ChIP-seq signal matrices were processed identically to the average profiles, including CNV correction and genome-wide mean subtraction. The rows of the resulting matrices were sorted randomly.

### Hi-C data processing

Sequencing reads were processed using the scsHi-C pipeline (Langer, 2021) to generate .pairs files (Lee et al, 2022) containing genomic contact coordinates. Replicate files were merged using pairtools v1.1.3 (Open2C et al, 2023). Following sister chromatid assignment, contacts were classified as cis sister, trans sister, or undefined, and separate .pairs files were generated for cis sister, trans sister, and all contacts. These files were converted into contact matrices at 1 kb resolution in cooler (Abdennur and Mirny, 2020) format using cooler v0.10.2 (Abdennur et al, 2025). The cis sister and trans sister matrices were summed to generate a combined sister map. All resulting matrices were aggregated into multiple resolutions. The combined sister and all-contact maps were balanced by iterative correction (Imakaev et al, 2012) with default parameters. Balancing weights derived from the combined sister maps were subsequently applied to the cis sister and trans sister maps.

To compare Hi-C map features across cell-cycle stages (Fig. 1A–C), the G2-synchronized and G1-synchronized contact maps were downsampled to match the total number of contacts in the G2-synchronized ΔSororin map and the individual G2-synchronized biological replicates.

### TAD calling and comparison of annotations

The hierarchy of topologically associated domains was annotated using OnTAD v1.2 (An et al, 2019) on scsHi-C maps of “all” contacts. To compare TAD architectures across cell cycle stages (Fig. 1C), TADs were called on downsampled contact maps at 20 kb resolution using parameters -minsz 2, -maxsz 200, and -penalty 1e-1. TAD annotations were compared using the adjusted Rand Index (ARI) (Hubert and Arabie, 1985; An et al, 2019). Briefly, each pixel in the contact map (up to 2 Mb genomic separation) was assigned the ID of the lowest-level TAD covering it. These pixel assignments were treated as clusters, and the similarity between clusterings from different maps was quantified using ARI.

To annotate hierarchical levels of TAD boundaries (Fig. 2 and onwards), TADs were called on the all-contact map of G2-synchronized wild-type HeLa cells at 10 kb resolution (-minsz 5, -maxsz 400, and -penalty 1e-1). To ensure robustness, the boundary list was filtered to exclude artifacts following the logic of the cooltools v0.7.1 (Open2C et al, 2024a) insulation method. Boundaries overlapping or adjacent to low-coverage bins were discarded. Additionally, boundaries were required to have at least 2/3 valid pixels in local insulation diamonds ( ± 50 kb, ±100 kb, and ±250 kb). Each valid boundary was assigned a hierarchical level corresponding to the highest level (smallest level number) of the TADs it demarcates. Boundaries were subsequently classified into three categories: high (level 1), mid (levels 2–3), and low (levels 4 + ).

### Stratification of TAD boundaries by compartment type

Compartmentalization was calculated from scsHi-C contact maps of wild-type G2-synchronized HeLa cells (50 kb resolution) (Mitter et al, 2020; Data ref: Mitter et al, 2020a) using “eigs_cis” function in cooltools v0.7.1 (Open2C et al, 2024a), utilizing hg19 GC-content as a phasing track. 50 kb genomic bins were assigned to A compartment if their first eigenvector (E1) value was positive, and to B compartment if negative. TAD boundaries were then assigned the compartment identity of the 50 kb bin in which they were located.

### Stratification of TAD boundaries by proximity to centromeres

Reference centromere coordinates for hg19 were obtained using bioframe v0.8.0 (“fetch_centromeres”) (Open2C et al, 2024b). Boundaries falling within a 10 Mb distance of any centromeric region were designated as centromere-proximal, while all other boundaries were assigned to chromosomal arms.

### Average Hi-C contact maps

Average Hi-C contact maps (pileups) centered at TAD boundaries were calculated using cooltools v0.7.1 (Open2C et al, 2024a). Briefly, contact submatrices centered on regions of interest were extracted and averaged pixel-wise. The first two diagonals were excluded to minimize Hi-C artifacts. To generate observed-over-expected pileups, the averaged observed contact frequencies were divided by the expected contact frequency (genome-wide average) for each genomic separation distance.

### Hi-C contact probability curves

Distance-dependent contact probability (*P(s)*) curves for cis and trans sister interactions were derived using pairtools. Contacts were aggregated into 72 geometrically spaced bins spanning genomic distances from 1 bp to 1 Gb. The contact frequency in each bin was normalized by the area of covered base pairs. To account for variation in sequencing depth, the cis and trans sister curves from each sample were scaled jointly, such that the total area under both curves summed to one across genomic separations of 10 kb to 200 Mb.

### Insulation score analysis

The insulation of cis and trans sister contacts was estimated using the insulation score, modified from (Wutz et al, 2017). Briefly, the score is calculated in sliding genomic windows as the negative log2 ratio of intra-window contacts spanning the window center to the total number of intra-window contacts. The resulting values are assigned to the window centers. Finally, to correct for differences in distance-decay effects (*P(s)*) between conditions, the genome-wide median is subtracted.

To profile the insulation landscape across cell-cycle stages (Fig. 1B), insulation scores were computed from downsampled Hi-C maps of “all” contacts at 20 kb resolution using a ±500 kb sliding window, excluding the first two diagonals. To profile insulation of sister-specific contacts in G2 phase, insulation scores were computed from full scsHi-C maps at 10 kb resolution using a ±500 kb sliding window. For cis sister insulation, the first two diagonals were excluded to minimize Hi-C artifacts. For trans sister insulation, the first 15 diagonals were excluded to mitigate the influence of short-range trans sister contact enrichment or depletion at boundaries. Insulation scores were saved as bigWig tracks using pybigtools v0.4.2 (Huey and Abdennur, 2024). Average profiles of insulation scores at TAD boundaries of different levels (Fig. 3 and onwards) were generated by aggregating genome-wide insulation score tracks in ±500 kb windows centered on TAD boundaries.

### Assignment of CTCF sites to TAD boundaries

To assign CTCF sites to TAD boundaries, boundary coordinates were expanded to capture the extended transition zones characteristic of mammalian genomes (Chang et al, 2020) and the associated clustering of CTCF sites (Chang et al, 2023). Specifically, boundary regions were defined as 50 kb intervals centered on the original boundary calls (±25 kb). This window size was selected as the maximum width that accommodates the extended boundary zone while ensuring that adjacent boundary regions remain non-overlapping. Reproducible CTCF peaks were then assigned to boundaries by intersecting peak coordinates with these expanded intervals.

### Statistical testing, data analysis, and visualization

Sequencing data of Hi-C experiments from CTCF-AID/SCC1-HALO, NIPBL-AID, and WAPL-dTAG cell lines without inducing target depletion were obtained from three technical replicates; all other sequencing data were obtained from at least two biological replicates for each condition. Statistical testing was restricted to peak calling. In box plots, the central line represents the median, the box limits indicate the first and third quartiles, and the whiskers extend to the 5th and 95th percentiles.

Analyses were performed in Python v3.10 using standard analysis packages (NumPy v1.25.2 (Harris et al, 2020), Pandas v1.5.3 (The pandas development team, 2023), SciPy v1.15.2 (Virtanen et al, 2020)). Data exploration and visualization were conducted using HiCognition (Langer et al, 2023), IGV (Thorvaldsdóttir et al, 2013; Robinson et al, 2011), HiGlass (Kerpedjiev et al, 2018) via resgen.io, Matplotlib v3.9.4 (Hunter, 2007), and seaborn v0.13.2 (Waskom, 2021).

### Declaration of generative AI and AI-assisted technologies in the manuscript preparation process

ChatGPT (version 5.2, OpenAI) was used to assist in improving the grammar of the manuscript text. No content was generated or modified relating to the scientific findings, data interpretation, or conclusions. All edits were reviewed and approved by the authors.

## Supplementary information

Peer Review File

Expanded View Figures

## Data Availability

The datasets and computer code produced in this study are available in the following databases: ChIP-seq data: ENA PRJEB108635 (https://www.ebi.ac.uk/ena/browser/view/PRJEB108635). Hi-C data: ENA PRJEB108635 (https://www.ebi.ac.uk/ena/browser/view/PRJEB108635). Processed sequencing data: BioStudies S-BSST2754 (https://www.ebi.ac.uk/biostudies/studies/S-BSST2754). Peakflow, ChIP-seq downstream processing pipeline: GitHub (https://github.com/gerlichlab/peakflow/tree/a61dd9678db3f46490bc195b8434a2f7e5722064). Reproducible JupyterLab environment: GitHub (https://github.com/gerlichlab/replchromconf-jupyterlab/tree/a357a7ee84a3e1385212aeaf81ab7144eafd3924). Processing scripts and analytical notebooks: GitHub (https://github.com/gerlichlab/takacs_mylarshchikov_et_al/tree/51e187e3cf40692ab81d410966f5792314febb54). The source data of this paper are collected in the following database record: biostudies:S-SCDT-10_1038-S44318-026-00902-0.
