# Supplementary material for: Nested TAD hierarchy defines cohesion zones on replicated chromosomes
Source: EMBO J. 2026 Aug 17;45(19):6883–913. doi: 10.1038/s44318-026-00902-0 (PMC13631365; doi:10.1038/s44318-026-00902-0)
Supplement: Supplementary file 1 [file 44318_2026_902_MOESM1_ESM.pdf]

THE EMBO JOURNAL

Nested TAD hierarchy defines cohesion zones on replicated chromosomes

Zsuzsanna Takacs, Dmitry Mylarshchikov, Sofia Kolesnikova, Christoph Langer, Anton Goloborodko, and Daniel Gerlich

Corresponding author(s): Daniel Gerlich (daniel.gerlich@imba.oeaw.ac.at) , Anton Goloborodko (anton.goloborodko@imba.oeaw.ac.at)

Review Timeline:

Submission Date:	9th Apr 26
Editorial Decision:	20th May 26
Revision Received:	17th Jul 26
Editorial Decision:	29th Jul 26
Revision Received:	2nd Aug 26
Accepted:	4th Aug 26

Editor: Hartmut Vodermaier

Transaction Report:

(Note: With the exception of the correction of typographical or spelling errors that could be a source of ambiguity, letters and reports are not edited. Depending on transfer agreements, referee reports obtained elsewhere may or may not be included in this compilation. Referee reports are anonymous unless the Referee chooses to sign their reports.)

Dr. Daniel W. Gerlich
Institute of Molecular Biotechnology of the Austrian Academy of Sciences (IMBA)
Dr. Bohrgasse 3
Vienna 1030
Austria

20th May 2026

Re: EMBOJ-2026-124333
Nested TAD hierarchy defines cohesion zones on replicated chromosomes

Dear Daniel,

Thank you for submitting your study dissecting distinct cohesin activities to The EMBO Journal, and apologies for the delay in getting back to you with a decision. We sent your study to three expert referees, but despite multiple reminders sent from our office, have still not received comments from one of them. Since the other two reports are in good agreement, I chose to contact you with a final decision now, in order to prevent unnecessary further loss of time.

As you will see, referees 2 and 3 consider your study both important and convincing, and recommend its publication pending adequate clarification of a few particular issues noted in their reports. I am therefore inviting you to incorporate these suggestions and to resubmit a revised manuscript, ideally already taking into account our specific guidelines for preparation/formatting of revisions and file uploading (as detailed below and in our Guide to Authors) - adhering to them as closely as possible shall greatly facilitate editorial processing upon resubmission. Should you have any questions in this regards, please do not hesitate to contact us directly.

Thank you again for the opportunity to consider this work for The EMBO Journal, and I look forward to your revision.

With kind regards,

Hartmut

Hartmut Vodermaier, PhD
Senior Editor, The EMBO Journal
h.vodermaier@embojournal.org

*** PLEASE NOTE: All revised manuscript are subject to initial checks for completeness and adherence to our formatting guidelines. Revisions may be returned to the authors and delayed in their editorial re-evaluation if they fail to comply to the following requirements. As a first step please read our guidelines for revised submissions:
https://link.springer.com/journal/44318/submission-guidelines#cms-Revised-submissions

1) Every manuscript requires a Data Availability section (even if only stating that no deposited datasets are included). Primary datasets or computer code produced in the current study have to be deposited in appropriate public repositories prior to resubmission, and reviewer access details provided in case that public access is not yet allowed.

2) Each figure legend must specify
- size of the scale bars that are mandatory for all micrograph panels
- the statistical test used to generate error bars and P-values
- the type error bars (e.g., S.E.M., S.D.)
- the number (n) and nature (biological or technical replicate) of independent experiments underlying each data point
- Figures may not include error bars for experiments with n<3; scatter plots showing individual data points should be used instead.

3) Revised manuscript text (including main tables, and figure legends for main and EV figures) has to be submitted as editable text file (e.g., .docx format). We encourage highlighting of changes (e.g., via text color) for the referees' reference.

4) Each main and each Expanded View (EV) figure should be uploaded as individual production-quality files (preferably in .eps, .tif, .jpg formats). For suggestions on figure preparation/layout, please refer to our Figure Preparation Guidelines:
https://media.springernature.com/original/springer-cms/rest/v1/content/27825798/data/v1

5) Point-by-point response letters should include the original referee comments in full together with your detailed responses to them (and to specific editor requests if applicable), and also be uploaded as editable (e.g., .docx) text files.

6) Please complete our Author Checklist, and make sure that information entered into the checklist is also reflected in the manuscript; the checklist will be available to readers as part of the Review Process File.

7) All authors listed as (co-)corresponding need to deposit, in their respective author profiles in our submission system, a unique ORCiD identifier linked to their name. Please see our Guide to Authors for detailed instructions.

8) Please note that supplementary information at EMBO Press has been superseded by the 'Expanded View' for inclusion of additional figures, tables, movies or datasets; with up to five EV Figures being typeset and directly accessible in the HTML version of the article.

9) To facilitate reproducibilty and cross-laboratory adoption of methodologies, please structure the Materials & Methods section as outlined in our guide to authors, including a completed Reagents and Tools Table.

10) Digital image enhancement is acceptable practice, as long as it accurately represents the original data and conforms to community standards. If a figure has been subjected to significant electronic manipulation, this must be clearly noted in the figure legend and/or the 'Materials and Methods' section. The editors reserve the right to request original versions of figures and the original images that were used to assemble the figure. Finally, we generally encourage uploading of numerical as well as gel/blot image source data.

At EMBO Press we ask authors to provide source data for the main manuscript figures. You will receive a separate email with instructions for providing source data with your revised manuscript, including how to upload and organize the files.

In the interest of ensuring the conceptual advance provided by the work, we recommend submitting a revision within 3 months (18th Aug 2026). Please discuss the revision progress ahead of this time with the editor if you require more time to complete the revisions. Use the link below to submit your revision:

Link Not Available

Referee #2:

In this study, Takacs et al. investigate how cells maintain 3D genome structure after DNA replication, a process essential for gene regulation and DNA repair. It resolves the apparent conflict between the two roles of cohesin: extruding DNA loops and holding sister chromatids together. Using cell cycle synchronization, G2-specific protein depletion, sister-chromatid-sensitive Hi-C, the authors show that these functions of cohesin are spatially separated within chromosomal domains. Cohesin that mediates sister-chromatid cohesion accumulates at major boundary regions, forming "cohesion zones." In contrast, cohesin responsible for dynamic loop extrusion operates at all boundaries. The authors propose that this segregation is facilitated by CTCF boundary elements, which act as semi-permeable barriers. This partitioning allows replicated chromosomes to simultaneously support the dynamic folding needed for transcription and the stable tethering required for accurate sister-chromatid-templated DNA repair.

Overall, the study addresses a major question in the chromatin field, i.e. how loop extruding cohesin cope with cohesive cohesin on replicated chromosomes in interphase. Experiments are well designed and performed to high technical quality. The paper is nicely written and a pleasure to read. Publication is recommended.

I only have two minor points that the authors may wish to address.

Minor points
(1) The authors should examine whether cohesin/sororin distributions differ between A and B compartments. Likewise, it will be interesting to check whether there are specific patterns in centromere-proximal regions.

(2) The authors should mention in the main text the types of cells they used for their study.

Referee #3:

The ATPase cohesin complex belongs to the SMC family and fulfills two distinct functions: it establishes sister chromatid cohesion by topologically entrapping sister DNAs, and it also promotes DNA loop formation through the process of loop

extrusion. In mammalian cells, cohesin-mediated loop extrusion is essential for partitioning the genome into topologically associated domains (TADs) and for regulating DNA-dependent processes during interphase. Nevertheless, it remains unclear whether, and how, these two distinct activities of cohesin interact and regulate one another during interphase. In the present study, Takacs et al. combine elegant approaches, including ChIP-seq, Hi-C, and sister-chromatid-sensitive Hi-C, to investigate how two distinct populations of cohesin are distributed within nested TADs. The authors show that cohesin involved in sister chromatid cohesion preferentially accumulates at high-level boundaries, whereas loop-extruding cohesin occupies boundaries across all hierarchical levels. Their findings suggest that the translocation of extruding cohesin displaces cohesion-associated cohesin, thereby confining it to high-level boundaries enriched in CTCF sites. Overall, the manuscript is clearly written, technically sound, and presents important and conceptually interesting findings. I would therefore recommend acceptance after the authors address the following minor points :

Figure 4A. If loop extrusion concentrates cohesion-associated cohesin at high-level boundaries, one would expect that NIPBL inactivation would not only impair sororin accumulation at high-level boundaries, but also promote its accumulation toward low- and middle-level boundaries. However, according to Figure 4A, NIPBL inactivation appears to reduce sororin occupancy at all loci. The authors should clarify how this observation fits with their proposed model.

Figures S3, S4, and S5. In all analyses, the authors compare wild-type and depleted conditions. Including an untreated tagged condition would strengthen the study by allowing a better assessment of the specific effects of protein depletion and by determining whether the tagged proteins themselves affect 3D genome organisation.

Takacs and Mylarshchikov et al. – response to the reviewers

We thank the reviewers for their thorough assessment of our work and the constructive suggestions how to improve the manuscript. We have addressed all concerns as explained point-by-point below.

Referee #2:

In this study, Takacs et al. investigate how cells maintain 3D genome structure after DNA replication, a process essential for gene regulation and DNA repair. It resolves the apparent conflict between the two roles of cohesin: extruding DNA loops and holding sister chromatids together. Using cell cycle synchronization, G2-specific protein depletion, sister-chromatid-sensitive Hi-C, the authors show that these functions of cohesin are spatially separated within chromosomal domains. Cohesin that mediates sister-chromatid cohesion accumulates at major boundary regions, forming "cohesion zones." In contrast, cohesin responsible for dynamic loop extrusion operates at all boundaries. The authors propose that this segregation is facilitated by CTCF boundary elements, which act as semi-permeable barriers. This partitioning allows replicated chromosomes to simultaneously support the dynamic folding needed for transcription and the stable tethering required for accurate sister-chromatid-templated DNA repair.

Overall, the study addresses a major question in the chromatin field, i.e. how loop extruding cohesin cope with cohesive cohesin on replicated chromosomes in interphase. Experiments are well designed and performed to high technical quality. The paper is nicely written and a pleasure to read. Publication is recommended.

I only have two minor points that the authors may wish to address.

Minor points

(1) The authors should examine whether cohesin/sororin distributions differ between A and B compartments. Likewise, it will be interesting to check whether there are specific patterns in centromere-proximal regions.

> We thank the reviewer for suggesting that we examine these regional chromosome features. We stratified TAD boundaries by their location within A- or B-compartment and, separately, by their location on chromosome arms or in centromere-proximal regions, and then analyzed Sororin and RAD21 distributions across the boundary hierarchy.
>
> The preferential enrichment of Sororin at high-level relative to low-level boundaries was preserved in both A and B compartments and in centromere-proximal regions, while RAD21 showed only modest variation across the boundary hierarchy, consistent with our genome-wide analysis. These new data, described in the Results and presented in Fig. S2A and B, corroborate our central conclusion that cohesive cohesin preferentially accumulates at high-level boundaries.

(2) The authors should mention in the main text the types of cells they used for their study.

> We thank the reviewer for pointing out this omission. All experiments in this study were performed in HeLa cells. We now state this explicitly when the experimental system is first introduced in the Results and provide the relevant details in the Methods.

Referee #3:

The ATPase cohesin complex belongs to the SMC family and fulfills two distinct functions: it establishes sister chromatid cohesion by topologically entrapping sister DNAs, and it also promotes DNA loop formation through the process of loop extrusion. In mammalian cells, cohesin-mediated loop extrusion is essential for partitioning the genome into topologically associated domains (TADs) and for

regulating DNA-dependent processes during interphase. Nevertheless, it remains unclear whether, and how, these two distinct activities of cohesin interact and regulate one another during interphase. In the present study, Takacs et al. combine elegant approaches, including ChIP-seq, Hi-C, and sister-chromatid-sensitive Hi-C, to investigate how two distinct populations of cohesin are distributed within nested TADs. The authors show that cohesin involved in sister chromatid cohesion preferentially accumulates at high-level boundaries, whereas loop-extruding cohesin occupies boundaries across all hierarchical levels. Their findings suggest that the translocation of extruding cohesin displaces cohesion-associated cohesin, thereby confining it to high-level boundaries enriched in CTCF sites. Overall, the manuscript is clearly written, technically sound, and presents important and conceptually interesting findings. I would therefore recommend acceptance after the authors address the following minor points :

Figure 4A. If loop extrusion concentrates cohesion-associated cohesin at high-level boundaries, one would expect that NIPBL inactivation would not only impair sororin accumulation at high-level boundaries, but also promote its accumulation toward low- and middle-level boundaries. However, according to Figure 4A, NIPBL inactivation appears to reduce sororin occupancy at all loci. The authors should clarify how this observation fits with their proposed model.

We thank the reviewer for this careful observation. We agree that NIPBL depletion reduces Sororin enrichment at all boundary classes rather than causing Sororin to accumulate at low- or mid-level boundaries (Fig. 4A,B). Instead, two observations indicate that Sororin-bound cohesin is redistributed away from boundary-centered peaks to become more broadly dispersed across chromatin.

First, chromatin fractionation shows that chromatin-bound Sororin remains high after NIPBL depletion (Fig. S4F), arguing against a general dissociation of Sororin-bound cohesin from chromatin. Second, the reduction in boundary-centered Sororin peaks is accompanied by an elevated baseline between boundary regions in the ChIP-seq tracks (Fig. 4A). Together, these findings indicate that the reduced Sororin signal at boundaries reflects spatial redistribution across chromatin rather than simple dissociation.

Consistent with this interpretation, Sororin enrichment becomes similar at low-, mid-, and high-level boundaries after NIPBL depletion (Fig. 4B). Thus, loss of loop extrusion abolishes the hierarchy-dependent distribution observed in wild-type cells: cohesive cohesin is no longer preferentially concentrated at high-level boundaries but is more broadly dispersed outside boundary-centered peaks. We have revised the Results to clarify this interpretation and to distinguish redistribution across chromatin from redistribution toward lower-level boundaries.

Figures S3, S4, and S5. In all analyses, the authors compare wild-type and depleted conditions. Including an untreated tagged condition would strengthen the study by allowing a better assessment of the specific effects of protein depletion and by determining whether the tagged proteins themselves affect 3D genome organisation.

We agree that untreated tagged controls are important for assessing baseline effects of the engineered cell lines. We have therefore added Hi-C contact maps from the untreated tagged lines alongside the wild-type and depletion conditions (Figs. S3J, S4E, and S5F). These data allow readers to directly evaluate the net baseline effects associated with each tagged line.

We retain wild-type cells as the primary reference because untreated degron-tagged cells can exhibit modest basal target degradation and/or tag-related effects and therefore do not necessarily represent an unperturbed state. Indeed, the untreated tagged lines show modest baseline differences in chromatin organization relative to wild type. Using wild type also provides a common reference across the different engineered lines and datasets. The depletion-associated phenotypes are substantially stronger than these baseline differences. We now explain this rationale in the revised manuscript.

Dr. Daniel W. Gerlich
Institute of Molecular Biotechnology of the Austrian Academy of Sciences (IMBA)
-
Dr. Bohrgasse 3
Vienna 1030
Austria

29th Jul 2026

Re: EMBOJ-2026-124333R
Nested TAD hierarchy defines cohesion zones on replicated chromosomes

Dear Daniel,

Thank you for submitting your revised manuscript for our consideration. I have now looked through the revision and your responses to the specific points raised by our two referees, and found the concerns well-addressed in the new version. We shall therefore be happy to accept the study for EMBO Journal publication, as soon as a few remaining editorial issues have been incorporated:

- Please list the contact email address for correspondence (i.e., your email) on the title page.

- Please carefully go through the reference list, which still contains several entries lacking full citation information such as volume or page/eLocator numbers.

- For preprint citations: Please adjust the in-text reference format according to "preprint: Miller et al, 2025"; and in the reference list, name of the platform plus DOI + preprint label - e.g. "bioRxiv doi: 1234/002.dfj123 [PREPRINT]". Please make sure that the Preprint label is only used for real preprints, not for papers that have already been formally published in a classical journal.

- As we have switched from a free-text author contribution statement towards a more formal statement based on Contributor Role Taxonomy (CRediT) terms, please remove the present Author Contribution section and instead specify each author's contribution(s) directly in the Author Information page of our submission system during upload of the final manuscript. See https://casrai.org/credit/ for more information.

- The currently separate declaration on AI use should become a sub-section at the end of the Methods section.

- Please double-check to make sure to all relevant funding information in the manuscript is congruent with the info entered into our submission system. At this stage, 'Austrian Academy of Sciences' is provided only in the Comments Box in the system - this should to be added as a separate entry via the 'More Funders' option; furthermore, 'Medical University of Vienna' is currently missing in the submission system.

- Please correct the nomenclature for the 'Supporting figures' to 'Figure EV1/2/3...' in all places: source file names, titles in the system, legends in the manuscript, references in the manuscript. SUPPLEMENTAL FIGURE LEGENDS should be updated to EXPANDED VIEW FIGURE LEGENDS.

- In the legends of figures 1B, C, please define the box plots in terms of minima, maxima, centre, bounds of box and whiskers, and percentile.

- Please provide suggestions for a short 'blurb' text prefacing and summing up the conceptual aspect of the study in two sentences (max. 250 characters), followed by 3-5 one-sentence 'bullet points' with brief factual statements of key results of the paper; they will form the basis of an editor-written 'Synopsis' accompanying the online version of the article. Please also upload a synopsis image, which can be used as a "visual title" for the synopsis section of your paper. The image should be in JPG format of 550 pixels wide and 300 pixels high, and please make sure that it remains clear enough even in these modest dimensions.

I am returning the manuscript to you for an ultimate round of minor revision, hoping you will be able to upload the modified final files by the end of this week, which would facilitate a swift proceeding with formal acceptance and production of the manuscript before the summer break.

With kind regards,

Hartmut

Hartmut Vodermaier, PhD
Senior Editor, The EMBO Journal
h.vodermaier@embojournal.org

*** PLEASE NOTE: All revised manuscript are subject to initial checks for completeness and adherence to our formatting guidelines. Revisions may be returned to the authors and delayed in their editorial re-evaluation if they fail to comply to the following requirements. As a first step please read our guidelines for revised submissions:
https://link.springer.com/journal/44318/submission-guidelines#cms-Revised-submissions

1) Every manuscript requires a Data Availability section (even if only stating that no deposited datasets are included). Primary datasets or computer code produced in the current study have to be deposited in appropriate public repositories prior to resubmission, and reviewer access details provided in case that public access is not yet allowed.

2) Each figure legend must specify
- size of the scale bars that are mandatory for all micrograph panels
- the statistical test used to generate error bars and P-values
- the type error bars (e.g., S.E.M., S.D.)
- the number (n) and nature (biological or technical replicate) of independent experiments underlying each data point
- Figures may not include error bars for experiments with n<3; scatter plots showing individual data points should be used instead.

3) Revised manuscript text (including main tables, and figure legends for main and EV figures) has to be submitted as editable text file (e.g., .docx format). We encourage highlighting of changes (e.g., via text color) for the referees' reference.

4) Each main and each Expanded View (EV) figure should be uploaded as individual production-quality files (preferably in .eps, .tif, .jpg formats). For suggestions on figure preparation/layout, please refer to our Figure Preparation Guidelines:
https://media.springernature.com/original/springer-cms/rest/v1/content/27825798/data/v1

5) Point-by-point response letters should include the original referee comments in full together with your detailed responses to them (and to specific editor requests if applicable), and also be uploaded as editable (e.g., .docx) text files.

6) Please complete our Author Checklist, and make sure that information entered into the checklist is also reflected in the manuscript; the checklist will be available to readers as part of the Review Process File.

7) All authors listed as (co-)corresponding need to deposit, in their respective author profiles in our submission system, a unique ORCiD identifier linked to their name. Please see our Guide to Authors for detailed instructions.

8) Please note that supplementary information at EMBO Press has been superseded by the 'Expanded View' for inclusion of additional figures, tables, movies or datasets; with up to five EV Figures being typeset and directly accessible in the HTML version of the article.

9) To facilitate reproducibilty and cross-laboratory adoption of methodologies, please structure the Materials & Methods section as outlined in our guide to authors, including a completed Reagents and Tools Table.

10) Digital image enhancement is acceptable practice, as long as it accurately represents the original data and conforms to community standards. If a figure has been subjected to significant electronic manipulation, this must be clearly noted in the figure legend and/or the 'Materials and Methods' section. The editors reserve the right to request original versions of figures and the original images that were used to assemble the figure. Finally, we generally encourage uploading of numerical as well as gel/blot image source data.

At EMBO Press we ask authors to provide source data for the main manuscript figures. You will receive a separate email with instructions for providing source data with your revised manuscript, including how to upload and organize the files.

In the interest of ensuring the conceptual advance provided by the work, we recommend submitting a revision within 3 months (27th Oct 2026). Please discuss the revision progress ahead of this time with the editor if you require more time to complete the revisions. Use the link below to submit your revision:

Link Not Available

EMBO Press Author Checklist

Corresponding Author Name: Daniel W. Gerlich	
Journal Submitted to: The EMBO Journal	
Manuscript Number: EMBOJ-2026-124333R	

USEFUL LINKS FOR COMPLETING THIS FORM
The EMBO Journal - Author Guidelines
EMBO Reports - Author Guidelines
Molecular Systems Biology - Author Guidelines
EMBO Molecular Medicine - Author Guidelines

Reporting Checklist for Life Science Articles (updated January
This checklist is adapted from Materials Design Analysis Reporting (MDAR) Checklist for Authors. MDAR establishes a minimum set of requirements in transparent reporting in the life sciences (see Statement of Task: 10.31222/osf.io/9sm4x). Please follow the journal's guidelines in preparing your
Please note that a copy of this checklist will be published alongside your article.

Abridged guidelines for figures
1. Data
The data shown in figures should satisfy the following conditions:
- ➞ the data were obtained and processed according to the field's best practice and are presented to reflect the results of the experiments in an accurate and unbiased manner.
- ➞ ideally, figure panels should include only measurements that are directly comparable to each other and obtained with the same assay.
- ➞ plots include clearly labeled error bars for independent experiments and sample sizes. Unless justified, error bars should not be shown for technical
- ➞ if n<5, the individual data points from each experiment should be plotted. Any statistical test employed should be justified.
- ➞ Source Data should be included to report the data underlying figures according to the guidelines set out in the authorship guidelines on Data

2. Captions
Each figure caption should contain the following information, for each panel where they are relevant:
- ➞ a specification of the experimental system investigated (eg cell line, species name).
- ➞ the assay(s) and method(s) used to carry out the reported observations and measurements.
- ➞ an explicit mention of the biological and chemical entity(ies) that are being measured.
- ➞ an explicit mention of the biological and chemical entity(ies) that are altered/varied/perturbed in a controlled manner.
- ➞ the exact sample size (n) for each experimental group/condition, given as a number, not a range;
- ➞ a description of the sample collection allowing the reader to understand whether the samples represent technical or biological replicates (including how many animals, litters, cultures, etc.).
- ➞ a statement of how many times the experiment shown was independently replicated in the laboratory.
- ➞ definitions of statistical methods and measures:
  - common tests, such as t-test (please specify whether paired vs. unpaired), simple χ2 tests, Wilcoxon and Mann-Whitney tests, can be unambiguously identified by name only, but more complex techniques should be described in the methods section;
  - are tests one-sided or two-sided?
  - are there adjustments for multiple comparisons?
  - exact statistical test results, e.g., P values = x but not P values < x;
  - definition of 'center values' as median or average;
  - definition of error bars as s.d. or s.e.m.

Please complete ALL of the questions below.
Select "Not Applicable" only when the requested information is not relevant for your study.

Materials

Newly Created Materials	Information included in the manuscript?	In which section is the information available? (Reagents and Tools Table, Materials and Methods, Figures, Data Availability Section)
New materials and reagents need to be available; do any restrictions apply?	Not Applicable	

Antibodies	Information included in the manuscript?	In which section is the information available? (Reagents and Tools Table, Materials and Methods, Figures, Data Availability Section)
For **antibodies** provide the following information:<br>- Commercial antibodies: RRID (if possible) or supplier name, catalogue number and or/clone number<br>- Non-commercial: RRID or citation	Yes	Reagents and Tools Table, Methods

DNA and RNA sequences	Information included in the manuscript?	In which section is the information available? (Reagents and Tools Table, Materials and Methods, Figures, Data Availability Section)
**Short novel DNA or RNA including primers, probes**: provide the sequences.	Not Applicable	

Cell materials	Information included in the manuscript?	In which section is the information available? (Reagents and Tools Table, Materials and Methods, Figures, Data Availability Section)
**Cell lines:** Provide species information, strain. Provide accession number in repository **OR** supplier name, catalog number, clone number, and/**OR** RRID.	Yes	Reagents and Tools Table, Methods
**Primary cultures:** Provide species, strain, sex of origin, genetic modification status.	Not Applicable	
Report if the cell lines were recently **authenticated** (e.g., by STR profiling) and tested for mycoplasma contamination.	Yes	Methods

Experimental animals	Information included in the manuscript?	In which section is the information available? (Reagents and Tools Table, Materials and Methods, Figures, Data Availability Section)
**Laboratory animals or Model organisms:** Provide species, strain, sex, age, genetic modification status. Provide accession number in repository **OR** supplier name, catalog number, clone number, **OR** RRID.	Not Applicable	
**Animal observed in or captured from the field:** Provide species, sex, and age where possible.	Not Applicable	
Please detail **housing and husbandry conditions**.	Not Applicable	

Plants and microbes	Information included in the manuscript?	In which section is the information available? (Reagents and Tools Table, Materials and Methods, Figures, Data Availability Section)
**Plants:** provide species and strain, ecotype and cultivar where relevant, unique accession number if available, and source (including location for collected wild specimens).	Not Applicable	
**Microbes:** provide species and strain, unique accession number if available, and source.	Not Applicable	

Human research participants	Information included in the manuscript?	In which section is the information available? (Reagents and Tools Table, Materials and Methods, Figures, Data Availability Section)
If collected and within the bounds of privacy constraints report on age, sex and gender or ethnicity for all study participants.	Not Applicable	

Core facilities	Information included in the manuscript?	In which section is the information available? (Reagents and Tools Table, Materials and Methods, Figures, Data Availability Section)
If your work benefited from core facilities, was their service mentioned in the acknowledgments section?	Yes	Acknowledgements

Design

Study protocol	Information included in the manuscript?	In which section is the information available? (Reagents and Tools Table, Materials and Methods, Figures, Data Availability Section)
If study protocol has been **pre-registered, provide DOI in the manuscript**. For clinical trials, provide the trial registration number **OR** cite DOI.	Not Applicable	
Report the **clinical trial registration number** (at ClinicalTrials.gov or equivalent), where applicable.	Not Applicable	

Laboratory protocol	Information included in the manuscript?	In which section is the information available? (Reagents and Tools Table, Materials and Methods, Figures, Data Availability Section)
Provide DOI OR other citation details if **external detailed step-by-step protocols** are available.	Not Applicable	

Experimental study design and statistics	Information included in the manuscript?	In which section is the information available? (Reagents and Tools Table, Materials and Methods, Figures, Data Availability Section)
Include a statement about **sample size** estimate even if no statistical methods were used.	Yes	Figure legends, Methods
Were any steps taken to minimize the effects of subjective bias when allocating animals/samples to treatment (e.g. **randomization procedure**)? If yes, have they been described?	Not Applicable	
Include a statement about **blinding** even if no blinding was done.	Not Applicable	
Describe **inclusion/exclusion criteria** if samples or animals were excluded from the analysis. Were the criteria pre-established?<br><br>If sample or data points were omitted from analysis, report if this was due to attrition or intentional exclusion and provide justification.	Yes	Methods
For every figure, are **statistical tests** justified as appropriate? Do the data meet the assumptions of the tests (e.g., normal distribution)? Describe any methods used to assess it. Is there an estimate of variation within each group of data? **I**s the variance similar between the groups that are being statistically compared?	Yes	Figure legends, Methods

Sample definition and in-laboratory replication	Information included in the manuscript?	In which section is the information available? (Reagents and Tools Table, Materials and Methods, Figures, Data Availability Section)
In the figure legends: state number of times the experiment was **replicated** in laboratory.	Yes	Figure legends
In the figure legends: define whether data describe **technical or biological replicates**.	Yes	Figure legends

Ethics

Ethics	Information included in the manuscript?	In which section is the information available? (Reagents and Tools Table, Materials and Methods, Figures, Data Availability Section)
Studies involving **human participants**: State details of **authority granting ethics approval** (IRB or equivalent committee(s), provide reference number for approval.	Not Applicable	
Studies involving **human participants**: Include a statement confirming that **informed consent** was obtained from all subjects and that the experiments conformed to the principles set out in the WMA Declaration of Helsinki and the Department of Health and Human Services Belmont Report.	Not Applicable	
Studies involving **human participants:** For publication of **patient photos**, include a statement confirming that consent to publish was obtained.	Not Applicable	
Studies involving experimental **animals**: State details of **authority granting ethics approval** (IRB or equivalent committee(s), provide reference number for approval. Include a statement of compliance with ethical regulations.	Not Applicable	
Studies involving **specimen and field samples:** State if relevant **permits** obtained, provide details of authority approving study; if none were required, explain why.	Not Applicable	

Dual Use Research of Concern (DURC)	Information included in the manuscript?	In which section is the information available? (Reagents and Tools Table, Materials and Methods, Figures, Data Availability Section)
Could your study fall under dual use research restrictions? Please check biosecurity documents and list of **select agents and toxins** (CDC): https://www.selectagents.gov/sat/list.htm	Not Applicable	
If you used a select agent, is the security level of the lab appropriate and reported in the manuscript?	Not Applicable	
If a study is subject to dual use research of concern regulations, is the name of the **authority granting approval and reference number** for the regulatory approval provided in the manuscript?	Not Applicable	

Reporting

The MDAR framework recommends adoption of discipline-specific guidelines, established and endorsed through community initiatives. Journals have their own policy about requiring specific guidelines and recommendations to complement MDAR.

Adherence to community standards	Information included in the manuscript?	In which section is the information available? (Reagents and Tools Table, Materials and Methods, Figures, Data Availability Section)
State if relevant guidelines or checklists (e.g., **ICMJE, MIBBI, ARRIVE, PRISMA**) have been followed or provided.	Not Applicable	
For **tumor marker prognostic studies,** we recommend that you follow the **REMARK** reporting guidelines (see link list at top right). See author guidelines, under 'Reporting Guidelines'. Please confirm you have followed these guidelines.	Not Applicable	
For **phase II and III randomized controlled trials**, please refer to the **CONSORT** flow diagram (see link list at top right) and submit the CONSORT checklist (see link list at top right) with your submission. See author guidelines, under 'Reporting Guidelines'. Please confirm you have submitted this list.	Not Applicable	

Data Availability

Data availability	Information included in the manuscript?	In which section is the information available? (Reagents and Tools Table, Materials and Methods, Figures, Data Availability Section)
Have **primary datasets** been deposited according to the journal's guidelines (see 'Data Deposition' section) and the respective accession numbers provided in the Data Availability Section?	Yes	Data Availability Section
Were **human clinical and genomic datasets** deposited in a public access-controlled repository in accordance to ethical obligations to the patients and to the applicable consent agreement?	Not Applicable	
Are **computational models** that are central and integral to a study available without restrictions in a machine-readable form? Were the relevant accession numbers or links provided?	Not Applicable	
If publicly available data were reused, provide the respective **data citations in the reference list**.	Yes	Data citations in the reference list
