# Supplementary material for: Nested TAD hierarchy defines cohesion zones on replicated chromosomes
Source: EMBO J. 2026 Aug 17;45(19):6883–913. doi: 10.1038/s44318-026-00902-0 (PMC13631365; doi:10.1038/s44318-026-00902-0)
Supplement: Supplementary file 2 [file 44318_2026_902_MOESM2_ESM.pdf]

Expanded View Figures

Figure EV1. Characterization of cohesin distribution in G1 and G2 cell cycle phases.

(**A**, **B**) Schematic of cell synchronization to G1 (**A**) and G2 (**B**) cell cycle phases. (**C–E**) Representative cell cycle profiles (phospho-H3S10 vs. DNA content) of asynchronous (**C**), G1-synchronized (**D**), and G2-synchronized (**E**) wild-type HeLa cells. The fraction of cells in the different cell cycle stages is shown. (**F**) RAD21 ChIP-Seq signal tracks (1 kb resolution) from G1-synchronized wild-type HeLa cells and RAD21, Sororin and CTCF ChIP-Seq tracks from G2-synchronized wild-type HeLa cells at the locus shown in Fig. 1A. Individual biological replicates are shown. RAD21 G1- and G2-synchronized replicates were downsampled to a matching sequencing depth (60 mln). ChIP-Seq data are CPM-normalized and centered by subtracting the genome-wide mean.

 © The Author(s)

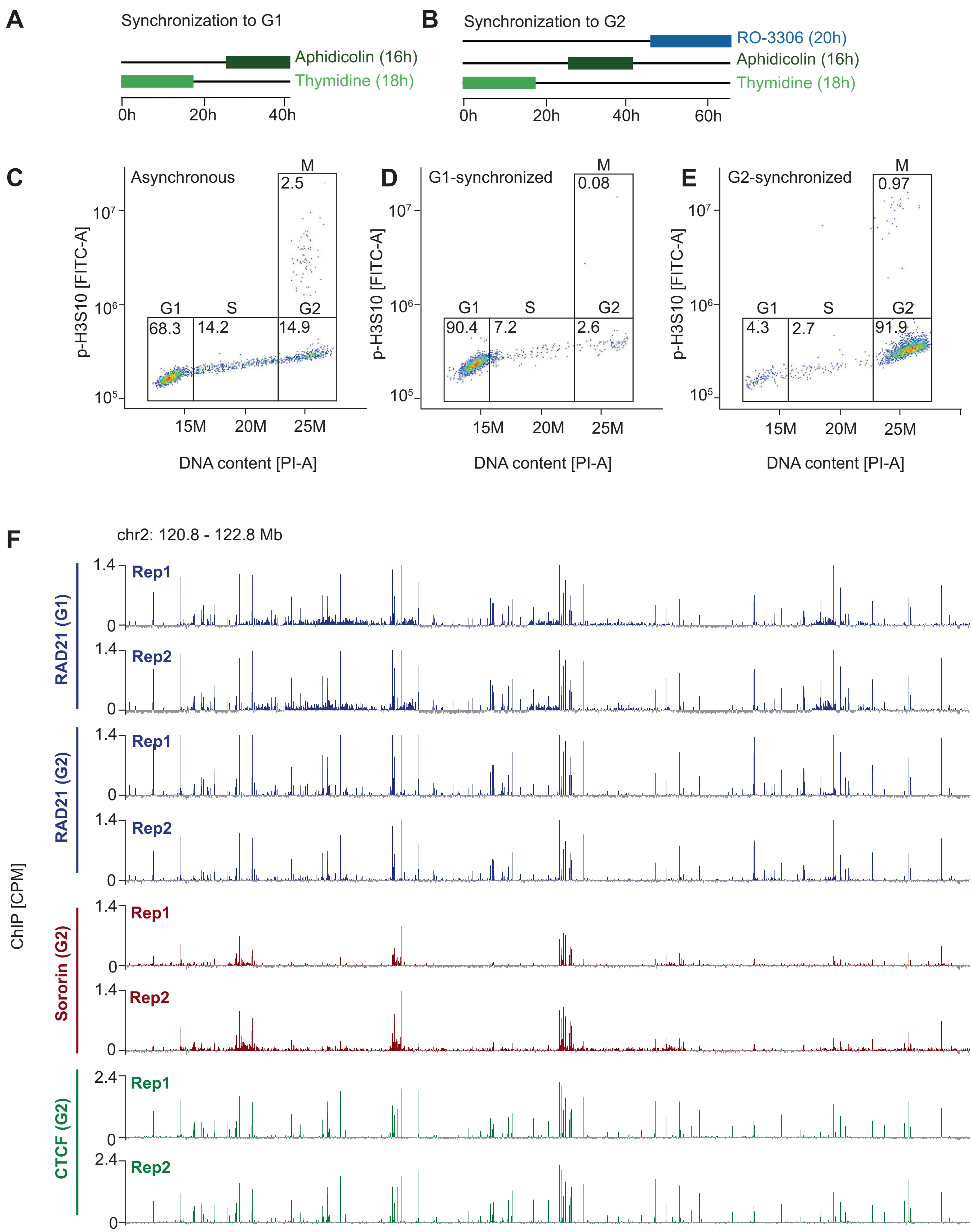
A
Synchronization to G1
Aphidicolin (16h)
Thymidine (18h)
0h
20h
40h
B
Synchronization to G2
RO-3306 (20h)
Aphidicolin (16h)
Thymidine (18h)
0h
20h
40h
60h
C
Asynchronous
M
2.5
G1
S
G2
68.3
14.2
14.9
p-H3S10 [FITC-A]
DNA content [PI-A]
15M
20M
25M
D
G1-synchronized
M
0.08
G1
S
G2
90.4
7.2
2.6
E
G2-synchronized
M
0.97
G1
S
G2
4.3
2.7
91.9
F
chr2: 120.8 - 122.8 Mb
ChIP [CPM]
RAD21 (G1)
Rep1
Rep2
RAD21 (G2)
Rep1
Rep2
Sororin (G2)
Rep1
Rep2
CTCF (G2)
Rep1
Rep2
1.4
2.4
0

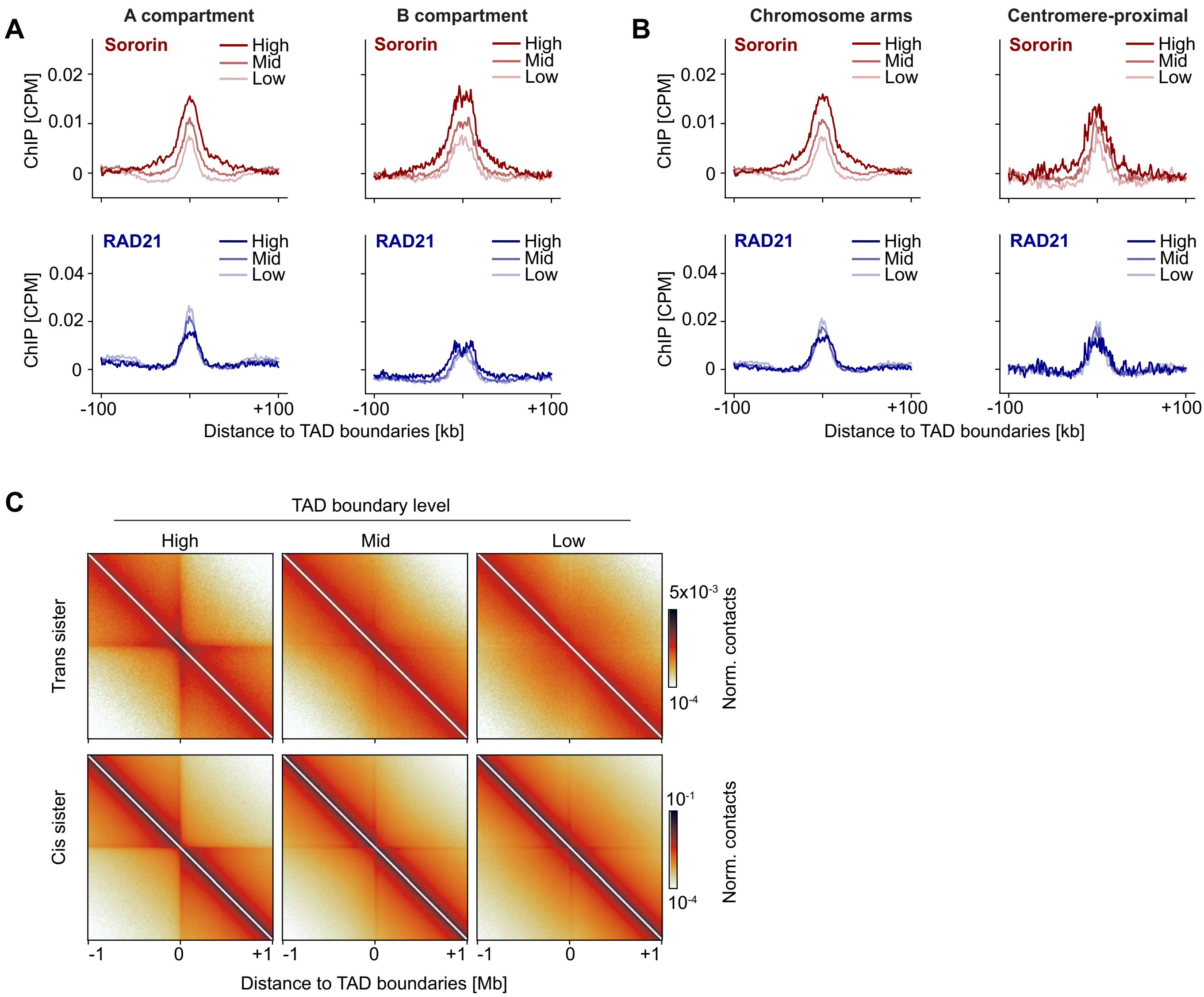

Figure EV2. Trans sister contacts accumulate at high-level boundaries.

(**A**) Average profiles of Sororin (upper row) and RAD21 (lower row) ChIP-Seq signal (1 kb resolution) at ±100 kb regions centered at high-, mid- and low-level boundaries within A (left column) and B compartments (right column). (**B**) Average profiles of Sororin (upper row) and RAD21 (lower row) ChIP-Seq signal (1 kb resolution) at ±100 kb regions centered at high-, mid- and low-level boundaries on chromosome arms (left column) and near centromeres (right column). (**C**) Average maps of trans sister (upper row) and cis sister (lower row) scsHi-C contacts (10 kb resolution) at ±1 Mb regions centered at high-, mid-, and low-level boundaries. Wild-type G2-synchronized HeLa scsHi-C data were reanalyzed from (Mitter et al, 2020; Data ref: Mitter et al, 2020a).

© The Author(s)

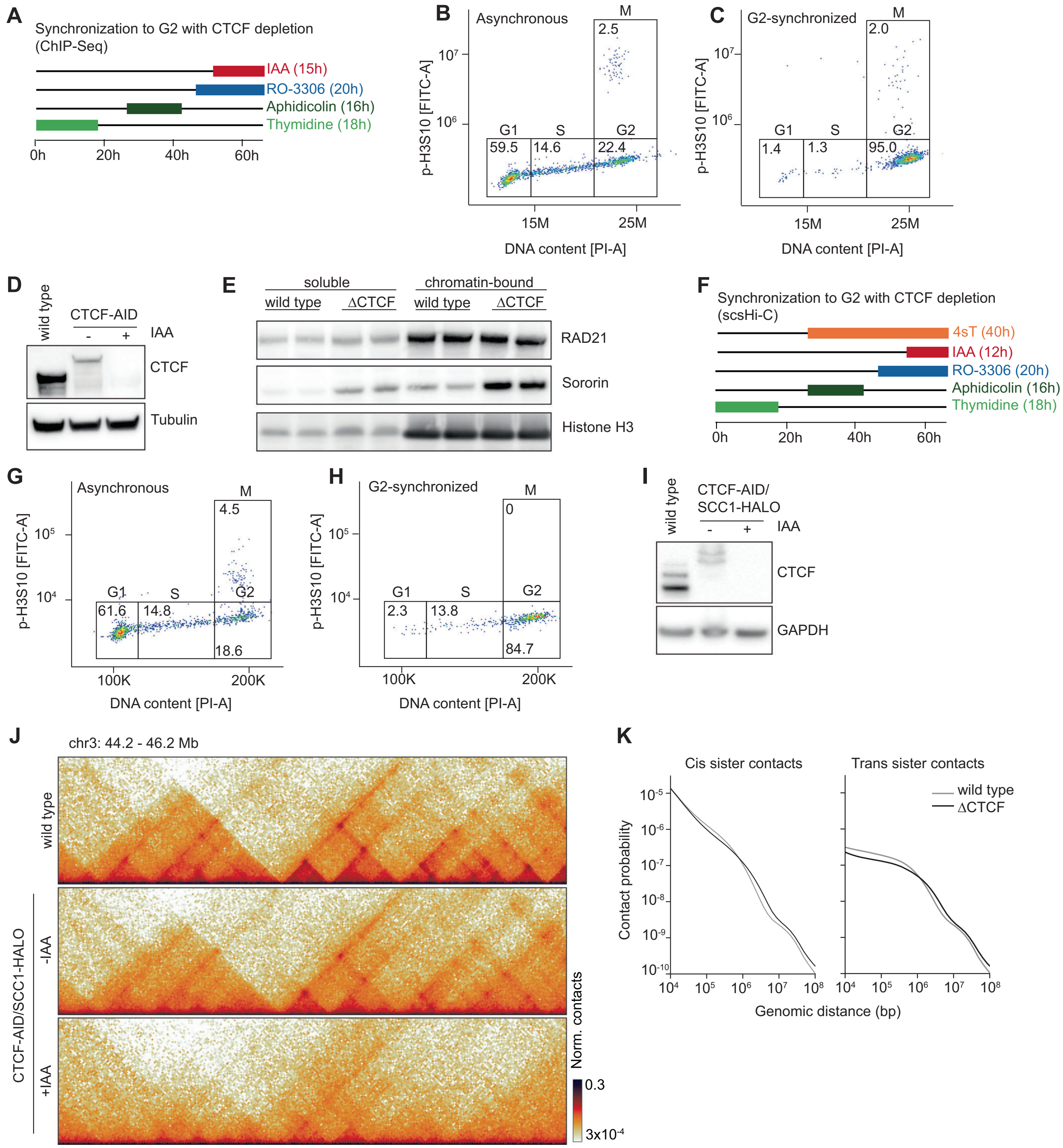
A
Synchronization to G2 with CTCF depletion (ChIP-Seq)
IAA (15h)
RO-3306 (20h)
Aphidicolin (16h)
Thymidine (18h)
0h 20h 40h 60h
B
Asynchronous
p-H3S10 [FITC-A]
DNA content [PI-A]
M 2.5
G1 59.5 S 14.6 G2 22.4
15M 25M
C
G2-synchronized
M 2.0
G1 1.4 S 1.3 G2 95.0
D
wild type
CTCF-AID
IAA
CTCF
Tubulin
E
soluble
chromatin-bound
wild type ΔCTCF wild type ΔCTCF
RAD21
Sororin
Histone H3
F
Synchronization to G2 with CTCF depletion (scsHi-C)
4sT (40h)
IAA (12h)
RO-3306 (20h)
Aphidicolin (16h)
Thymidine (18h)
G
Asynchronous
M 4.5
G1 61.6 S 14.8 G2 18.6
100K 200K
H
G2-synchronized
M 0
G1 2.3 S 13.8 G2 84.7
I
wild type
CTCF-AID/ SCC1-HALO
IAA
CTCF
GAPDH
J
chr3: 44.2 - 46.2 Mb
wild type
CTCF-AID/SCC1-HALO
-IAA
+IAA
Norm. contacts
0.3
3x10-4
K
Cis sister contacts
Trans sister contacts
wild type
ΔCTCF
Contact probability
Genomic distance (bp)

Figure EV3. Characterization of CTCF depletion for scsHi-C and ChIP-Seq experiments.

(**A**) Schematic of G2 cell synchronization and CTCF depletion for ChIP-Seq. (**B**, **C**) Representative cell cycle profiles (phospho-H3S10 vs. DNA content) of asynchronous (**B**) and G2-synchronized (**C**) HeLa CTCF-AID cells corresponding to (**A**). The fraction of cells in the different cell cycle stages is shown. (**D**) Western blot confirming CTCF depletion upon indole-3-acetic acid (IAA) treatment in G2-synchronized CTCF-AID cells corresponding to (**A**–**C**). Tubulin serves as a loading control. (**E**) Western blot confirming the presence of RAD21 and Sororin on chromatin in G2-synchronized CTCF-depleted (ΔCTCF, CTCF-AID) cells, as shown in (**A**, **C**). Histone H3 serves as a loading control. Data were representative of $n = 2$ independent experiments. (**F**) Schematic of G2 cell synchronization and CTCF depletion for scsHi-C. (**G**, **H**) Representative cell cycle profiles (phospho-H3S10 vs. DNA content) of asynchronous (**G**) and G2-synchronized (**H**) CTCF-AID/SCC1-HALO cells corresponding to (**F**). The fraction of cells in the different cell cycle stages is shown. (**I**) Western blot confirming CTCF depletion upon indole-3-acetic acid (IAA) treatment in G2-synchronized CTCF-AID/SCC1-HALO cells corresponding to (**F**–**H**). GAPDH serves as a loading control. (**J**) Representative Hi-C contact maps (10 kb resolution, 1 Mb separation) from wild-type cells, and CTCF-AID/SCC1-HALO cells in the presence and absence of indole-3-acetic acid (IAA). (**K**) Distance-dependent contact probability (P(s)) curves for cis and trans sister scsHi-C-contacts from G2-synchronized wildtype and CTCF-depleted (ΔCTCF, CTCF-AID/SCC1-HALO) cells. scsHi-C data from G2-synchronized CTCF-AID/SCC1-HALO cells without target depletion (-IAA) and upon CTCF depletion (ΔCTCF, +IAA) are merged from $n = 3$ technical replicates and $n = 5$ independent experiments, respectively. Wild-type G2-synchronized scsHi-C data were reanalyzed from (Mitter et al, 2020; Data ref: Mitter et al, 2020a).

 © The Author(s)

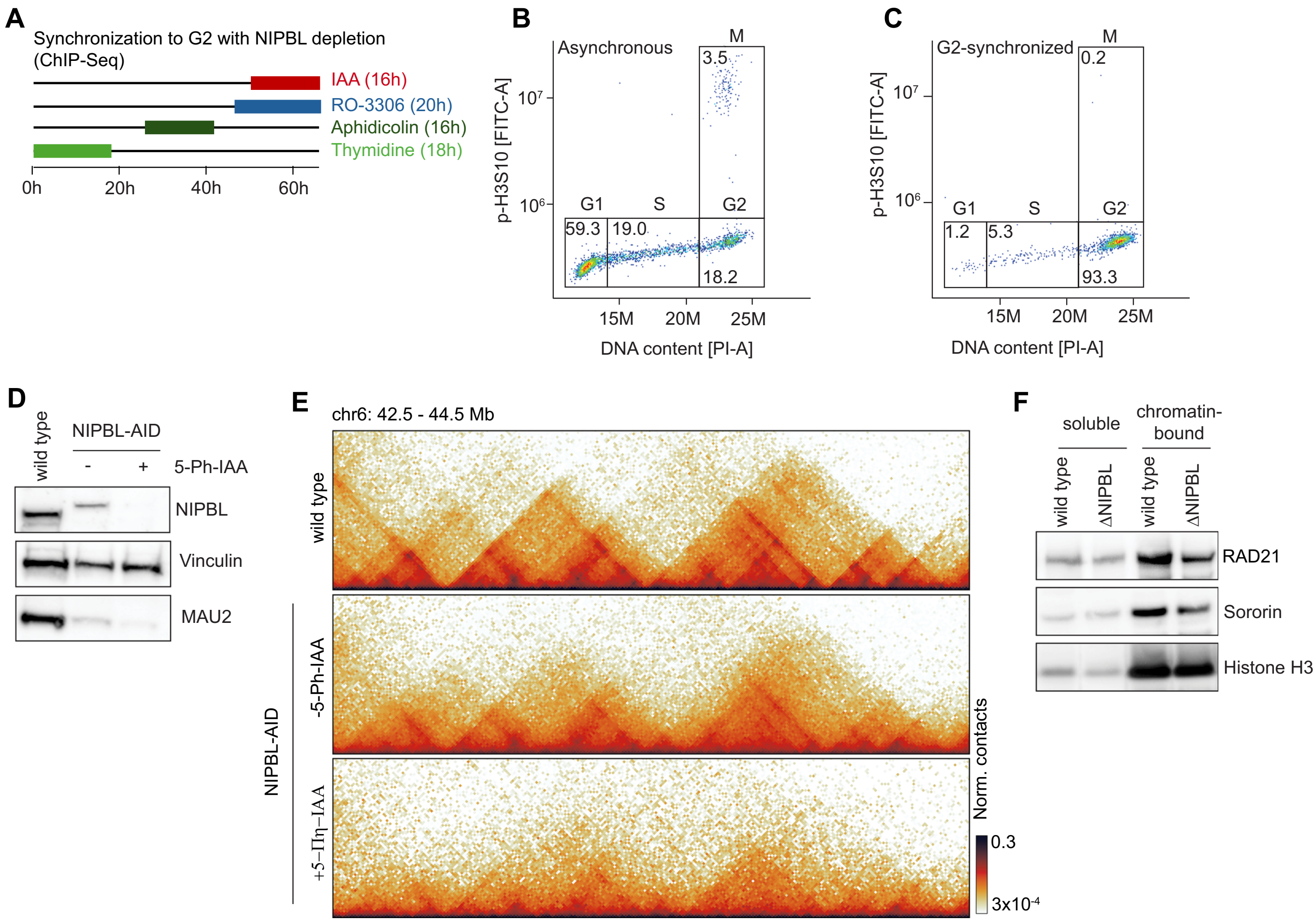

Figure EV4. Characterization of NIPBL depletion for ChIP-Seq experiments.

(**A**) Schematic of G2 synchronization and NIPBL depletion for ChIP-Seq. (**B**, **C**) Representative cell cycle profiles (phospho-H3S10 vs. DNA content) of asynchronous (**B**) and G2-synchronized (**C**) HeLa NIPBL-AID cells corresponding to (A). The fraction of cells in the different cell cycle stages is shown. (**D**) Western blot confirming NIPBL depletion upon 5-phenyl-indole-3-acetic acid (5-Ph-IAA) treatment in G2-synchronized NIPBL-AID cells corresponding to (**A**–**C**). NIPBL depletion was additionally confirmed by immunoblotting for its binding partner MAU2 (Watrin and Peters, 2006; Banigan et al, 2023). Vinculin serves as a loading control. (**E**) Representative Hi-C contact maps (10 kb resolution, 1 Mb separation) from G2-synchronized wild-type cells, and NIPBL-AID cells in the presence and absence of 5-phenyl-indole-3-acetic acid (5-Ph-IAA). (**F**) Western blot confirming the presence of RAD21 and Sororin on chromatin in G2-synchronized NIPBL-depleted (ΔNIPBL, NIPB-AID) cells, as shown in (**A**, **C**). Histone H3 serves as a loading control. Data are representative of $n = 2$ independent experiments. scsHi-C data from G2-synchronized NIPBL-AID cells without target depletion (-5-Ph-IAA) are merged from $n = 3$ technical replicates. Wild-type G2-synchronized scsHi-C data were reanalyzed from (Mitter et al, 2020; Data ref: Mitter et al, 2020a). ΔNIPBL G2-synchronized scsHi-C data were reanalyzed from (Batty et al, 2023; Data ref: Batty et al, 2023).

© The Author(s)

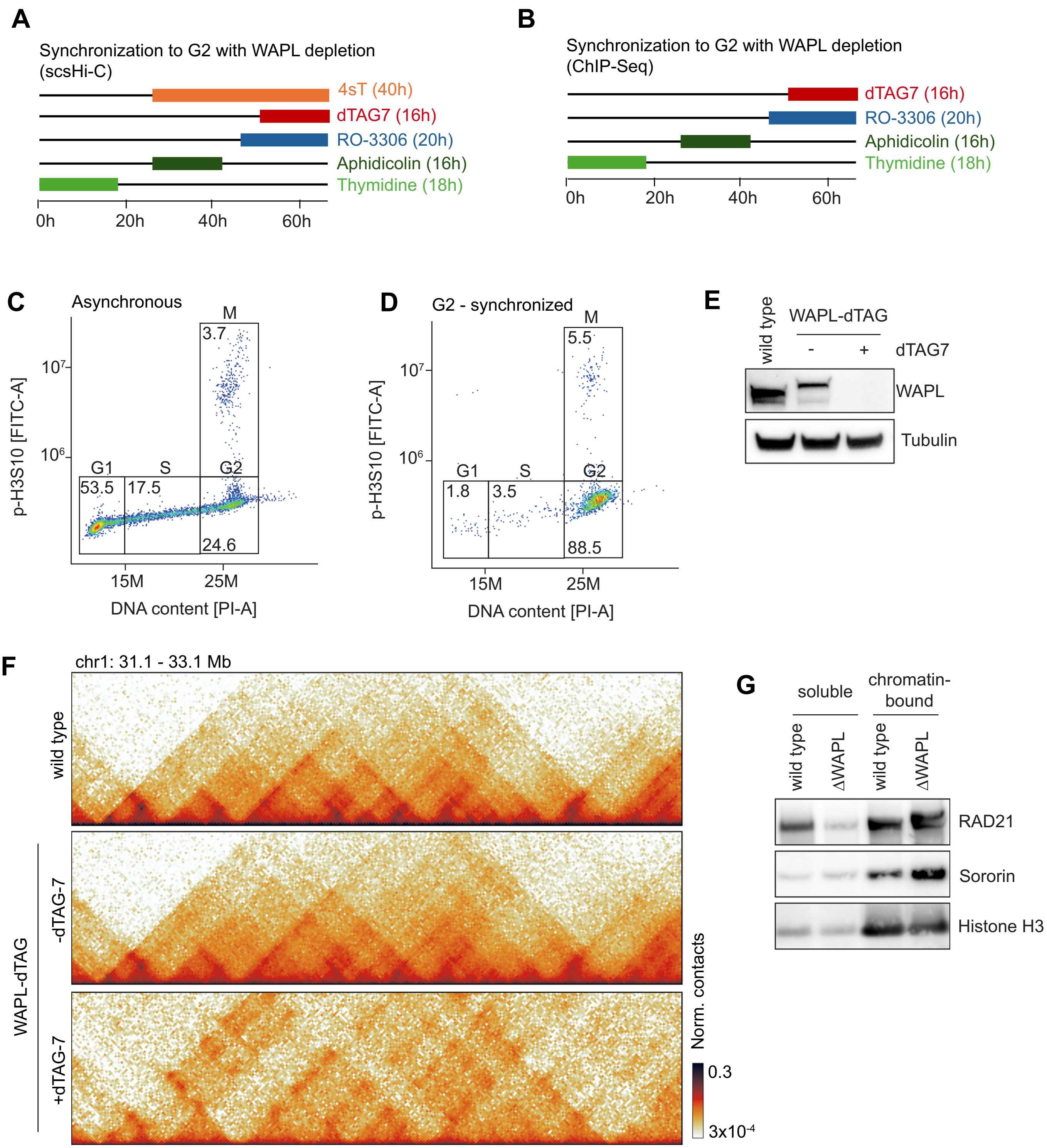

Figure EV5. Characterization of WAPL depletion for scsHi-C and ChIP-Seq experiments.

(**A**, **B**) Schematic of G2 synchronization and WAPL depletion for scsHi-C (**A**) and ChIP-Seq (**B**). (**C**, **D**) Representative cell cycle profiles (phospho-H3S10 vs. DNA content) of asynchronous (**C**) and G2-synchronized (**D**) HeLa WAPL-dTAG cells corresponding to (**A**, **B**). The fraction of cells in the different cell cycle stages is shown. (**E**) Western blot confirming WAPL depletion upon dTAG-7 treatment in G2-synchronized WAPL-dTAG cells corresponding to (**A**, **B**, **D**). Tubulin serves as a loading control. (**F**) Representative Hi-C contact maps (10 kb resolution, 1 Mb separation) from G2-synchronized wild type cells, and WAPL-dTAG cells in the presence and absence of dTAG-7. (**G**) Western blot confirming the presence of RAD21 and Sororin on chromatin in G2-synchronized WAPL-depleted (ΔWAPL and WAPL-dTAG) cells, as shown in (**B**, **D**). Histone H3 serves as a loading control. scsHi-C data from G2-synchronized WAPL-dTAG cells without target depletion (-dTAG-7) and upon WAPL depletion (ΔWAPL, +dTAG-7) are merged from $n = 3$ technical replicates and $n = 2$ independent experiments, respectively. Wild-type G2-synchronized scsHi-C data were reanalyzed from (Mitter et al, 2020; Data ref: Mitter et al, 2020a).

 © The Author(s)

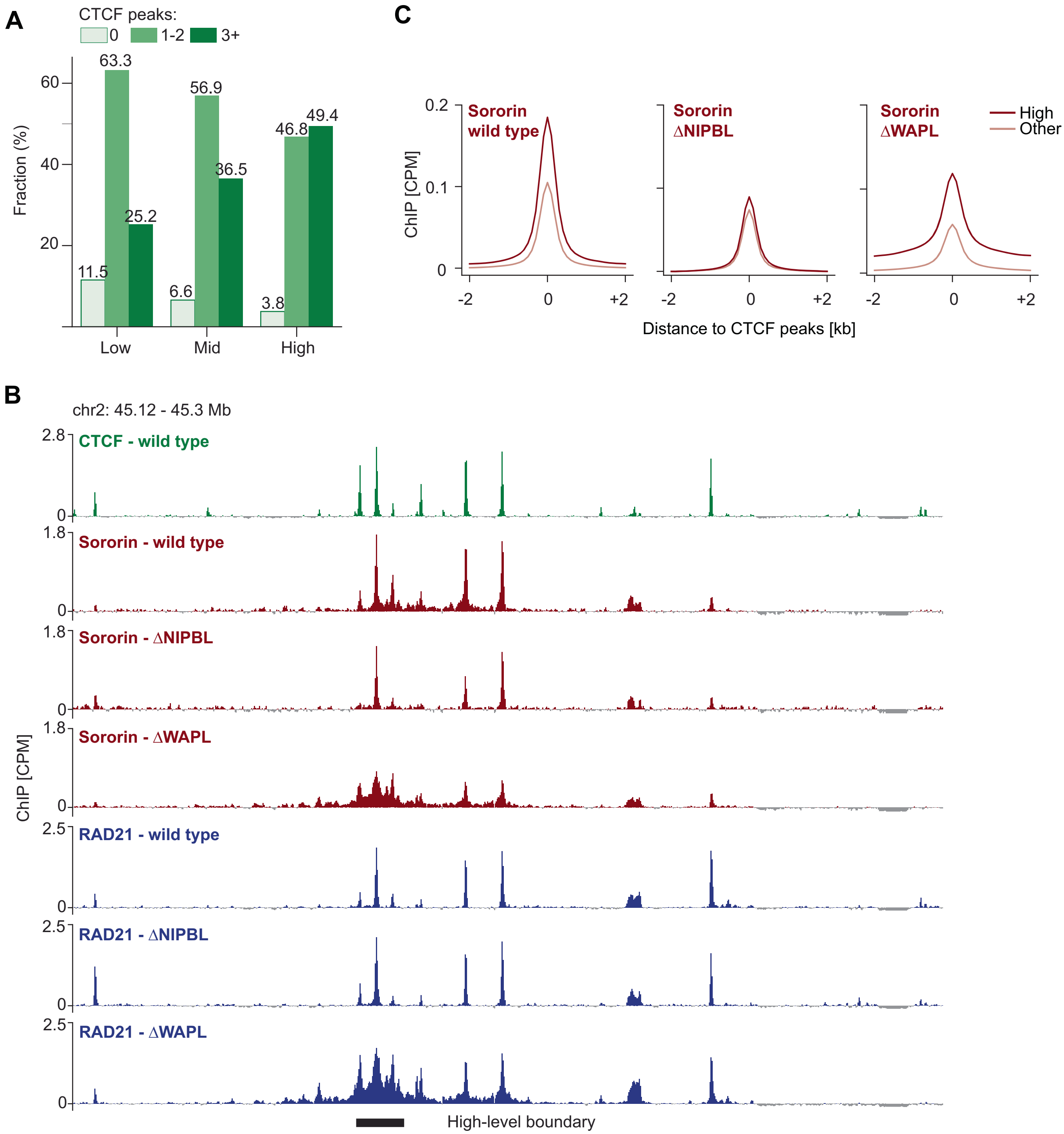

Figure EV6. Characterization of CTCF clusters at high-level boundaries.

(**A**) Fractions of low-, mid- and high-level boundaries containing 0, 1, 2, or ≥3 CTCF peaks. (**B**) Representative ChIP-Seq tracks (200 bp resolution) of CTCF (green) from G2-synchronized wildtype, and Sororin (red) and RAD21 (blue) from G2-synchronized wild type, NIPBL-depleted (ΔNIPBL, NIPBL-AID) and WAPL-depleted (ΔWAPL, WAPL-dTAG) HeLa cells. Position of a high-level boundary (10 kb) is indicated below. (**C**) Average profile of Sororin ChIP-Seq signal (100 bp resolution) in G2-synchronized cells under wild type (left panel), NIPBL-depleted (ΔNIPBL, middle panel) and WAPL-depleted (ΔWAPL, right panel) conditions at ±2 kb regions centered on isolated CTCF peaks (no additional CTCF peak within ±2.5 kb) and stratified by location: high-level boundaries (High) ($n = 5989$ peaks) or elsewhere in the genome (Other) ($n = 44,600$ peaks). ChIP-seq data were CPM-normalized, centered by subtracting the genome-wide mean and represent merged data from $n = 3$ (CTCF) or $n = 2$ (Sororin, RAD21) independent experiments.

© The Author(s)
